# Metabolomics and lipidomics studies of parasitic helminths: molecular diversity and identification levels achieved by using different characterisation tools

**DOI:** 10.1007/s11306-023-02019-5

**Published:** 2023-06-25

**Authors:** Phurpa Wangchuk, Karma Yeshi, Alex Loukas

**Affiliations:** Centre for Molecular Therapeutics, Australian Institute of Tropical Health and Medicine, James Cook University, Cairns, QLD 4878 Australia

**Keywords:** Metabolomics, Lipidomics, Helminths, Parasites

## Abstract

**Introduction:**

Helminths are parasitic worms that infect millions of people worldwide and secrete a variety of excretory-secretory products (ESPs), including proteins, peptides, and small molecules. Despite this, there is currently no comprehensive review article on cataloging small molecules from helminths, particularly focusing on the different classes of metabolites (polar and lipid molecules) identified from the ESP and somatic tissue extracts of helminths that were studied in isolation from their hosts.

**Objective:**

This review aims to provide a comprehensive assessment of the metabolomics and lipidomics studies of parasitic helminths using all available analytical platforms.

**Method:**

To achieve this objective, we conducted a meta-analysis of the identification and characterization tools, metabolomics approaches, metabolomics standard initiative (MSI) levels, software, and databases commonly applied in helminth metabolomics studies published until November 2021.

**Result:**

This review analyzed 29 studies reporting the metabolomic assessment of ESPs and somatic tissue extracts of 17 helminth species grown under ex vivo/in vitro culture conditions. Of these 29 studies, 19 achieved the highest level of metabolite identification (MSI level-1), while the remaining studies reported MSI level-2 identification. Only 155 small molecule metabolites, including polar and lipids, were identified using MSI level-1 characterization protocols from various helminth species. Despite the significant advances made possible by the ‘omics’ technology, standardized software and helminth-specific metabolomics databases remain significant challenges in this field. Overall, this review highlights the potential for future studies to better understand the diverse range of small molecules that helminths produce and leverage their unique metabolomic features to develop novel treatment options.

## Introduction

Helminths are classified into two major phyla: nematodes (or roundworms) and platyhelminths (or flatworms). Nematodes include intestinal worms, also known as soil-transmitted helminths, such as hookworms, roundworms, whipworms, and filarial worms. Platyhelminths include flukes or trematodes, such as schistosomes, and tapeworms, also known as cestodes (the pork tapeworm). These helminths are estimated to infect one-third of the almost three billion people in developing regions of Africa, Asia, and the Americas (Hotez et al., 2008). Intestinal nematodes alone infect approximately 24% of the world’s population, mainly affecting children in tropical and sub-tropical regions (WHO, 2020).

The intestinal nematodes, such as *Necator americanus, Ancylostoma duodenale, Ascaris lumbricoides*, and *Trichuris trichiura*, are major contributors to public health burden, causing anaemia, digestive diseases, and stunted growth (Sanchez et al., 2014). Despite their significant impact on global health burden, helminth infections are neglected tropical diseases and receive only 0.1% of global research funding (Moran, 2011). Currently, no vaccine is available for helminth infections, and the limited number of anthelmintic drugs, coupled with drug resistance in livestock animal nematodes, hinders the global fight against parasitic worms. Moreover, the gold standard for diagnosing helminth infections based on microscopy is labour-intensive and is less sensitive. PCR-based molecular diagnostic is expensive and the resource-constrained developing tropical countries cannot afford them. Therefore, there is a need for more sensitive, specific, and affordable diagnostic tools that can be used in these settings.

Helminth excretory/secretory products (ESPs) offer a promising source of biomarkers and immunomodulatory biomolecules, which are produced through unique biochemical pathways that have evolved over millennia of co-evolution between the parasites and humans (Loukas et al., 2016). The ESPs contain a variety of components, including extracellular vesicles, proteins, peptides, glycans, and small molecules, that has specific biological functions related to moulting, infection, immunopathogenesis, immunoregulation, reproduction, intra- and inter- specific competitions, and colony establishment in the gut (Eichenberger et al., 2018). Recently, the immunomodulatory and therapeutic potential of helminth-derived small molecules has been reviewed by Yeshi et al*.* (2022). Proteomics and glycomics techniques have been used to extensively study helminth-derived proteins, peptides, and glycans, and these studies have been reviewed elsewhere (Eichenberger et al., 2018; Wangchuk et al., 2019c).

Studies of helminth small molecules, particularly those focused on ESPs of intestinal parasites (i.e., removed from their hosts), are less advanced than transcriptomics and proteomics studies. This could be partly due to the lack of suitable culture media for gathering small molecule ESPs and complex identification protocols. The classical culture media such as RPMI-1640 contains more than 40 small molecules, hindering the identification of ESPs small molecule metabolites released in the culture media (Maizels et al., 2018). However, a simple single-component alternative culture media has been described recently, resolving the technical difficulties presented by RPMI culture media (McSorley et al., 2013; Wangchuk et al., 2019c). The recent development of cutting-edge analytical equipment and metabolomics bioinformatics platforms has enabled the investigation of small molecules produced by helminths, thus gaining attention.

Metabolomics is a technique used to characterize a complex mixture or a large number of metabolites or small molecules (< 1 kDa) that are both exogenous and endogenous and are produced by or present in an organism (Zakeri et al., 2018). In helminthology, metabolomics is a relatively new technique wiith great potential to identify metabolites in the cells, biofluids (including ESPs), tissues, and whole organisms (Ryan et al., 2020). Four major types of analytical equipment are widely used in metabolomics: gas chromatography-mass spectrometry (GC–MS), liquid chromatography-mass spectrometry (LC–MS), capillary electrophoresis-mass spectrometry (CE-MS), and nuclear magnetic resonance (NMR) spectroscopy (Perez de Souza et al., 2021). A review by Preidis and Hotez (Preidis & Hotez, 2015) discussed the metabolomic profiles of parasitised hosts and the potential of metabolomics techniques, including NMR, GC–MS, and LC–MS, to identify biomarkers for developing more sensitive point-of-care diagnostics for neglected tropical diseases. Kokova and Mayboroda (Kokova & Mayboroda, 2019) described the state-of-the-art NMR metabolomics of body fluids of animals or hosts infected with parasitic helminths, and Whitman et.al (Whitman et al., 2021) provided a comprehensive review of the metabolomics of parasitic helminths using mass spectrometry. To the best of our knowledge, a comprehensive review on the cataloguing of small molecules of helminths, particularly different classes of metabolites (polar and lipid molecules) identified from the ESPs and somatic tissue extracts of helminths collected in vitro after removal from their hosts, has not been attempted so far.

Herein, we have conducted a meta-analysis of the published information on small molecules of helminths collected from various databses, including PubMed, Embase, Scopus, Web of Science, and Google Scholar. We have collated all the available data and presented metabolites identified with the highest level of the Metabolomics Standards Initiative (MSI).We retrieved 28 metabolomics studies (involving 17 different helminth species) that used various analytical platforms and identification tools, and conducted a meta-analysis of these 28 papers. Table 1 summarises analytical equipment, databases and software used for metabolomics studies of various samples prepared from different developmental stages of helminths. We found that these 28 metabolomics studies used GC-MS, LC-MS, CE-MS, and NMR for studying the ESP and the somatic tissue extracts of various helminths. Besides these, a few helminth metabolomics studies included in this review have also applied other analytical platforms, including Raman and Fourier transform infrared (FTIR) spectroscopies, high-resolution mass spectrometry (HRM) such as Q-Exactive Orbitrap MS/HPLC, ultrahigh performance liquid-chromatography mass spectrometry (UHPLC/MS), and atmospheric pressure (AP) matrix-assisted laser desorption/ionization mass spectrometry imaging (AP-SMALDI MSI) (Table 1). The advantages and limitations of various mass spectrometry techniques have been reviewed in-depth elsewhere (Dettmer et al., 2007).

**Table 1:** Analytical techniques, databases, and software used for metabolomics studies of helminths

Phylum	Order	Helminth species	Stage	Sample	Culture media	Approaches/ design	Identification level	Number of metabolites	Analytical platform	Software(s)/databases used for metabolites identification	Reference
Nematoda	Trichocephalida	*Trichuris muris*	Embryonated eggs	SE	2% glutamax, 2% pen/strep PBS	Untargeted metabolomics	MSI-1 (polar) MSI-2 (lipids)	113 polar metabolites & 322 lipids	Q-Exactive Orbitrap MS-HPLC	KEGG, MetaCyc, LIPIDMAPS, PubChem CID, HMDB, CTS databases; open-source software IDEOM; MetaboAnalyst 3.0	(Yeshi et al., 2020)
			Adult	ESPs	2% glutamax, 2% pen/strep PBS	Targeted metabolomics	MSI-1	35 polar metabolites & 16 lipids	GC–MS	Agilent’s Mass Hunter Quantitative Analysis software (v.7); MAML; MHL; KEGG; MetaboAnalyst 3.0	(Wangchuk et al., 2019b)
		*Trichinella papuae*	L1 (muscle-stage)	SE	NA	Untargeted lipidomic	MSI-2	403 lipids	UPLC-MS/MS/ Xevo G2-XS QTof MS	LIPIPMAPS and LipidBlast for lipid identification; QuickGO – for gathering genes involved in lipid metabolism	(Mangmee et al., 2020)
	Ascaridida	*Ascaris suum*	L3, L4, adult	SE	NA	Untargeted lipidomic	MSI-2	587 lipids	UHPLC-MS/MS	LipidSearch software v.4.2.23; Intervene – to generate lipidomics UpSet plot	(Wang et al., 2020)
		*Ascaris lumbricoides*	Adult	ESPs	Modified Ringer-Locke’s solution	Targeted lipidomic	MSI-1	9 lipids	GLC	Comparing retention times of unknowns with standards	(Greichus & Greichus, 1966)
			Eggs, L1, L3	SE	NA	Fingerprint analysis (biomarkers)	MSI-2	9 biomarkers	HRMS	Lipid MAPS, HMDB (v 3.6), METLIN	(Melo et al., 2016)
		*Toxocara canis*	Adult	ESPs	2% Glutamax, 2% pen/strep PBS	Targeted metabolomics	MSI-1	41 polar metabolites & 20 lipids	GC–MS; LC–MS	Agilent’s Mass Hunter Quantitative Analysis software (v.7), MAML; MetaboAnalyst 3.0	(Wangchuk et al., 2020)
			Adult	SE	Eagle’s minimum essential medium (GIBCO)	Untargeted metabolomics	MSI-1	6 polar metabolites & 3 lipids	^1^H NMR	NA	(Learmonth et al., 1987)
	Spirurida	*Brugia malayi*	Adult	Cuticle	NA	Targeted lipidomic	MSI-1	17 lipids	TLC, GC	Comparing retention times of unknowns with authentic standards	(Smith et al., 1996)
	Strongylida	*Haemonchus contortus*	Eggs, L3, xL3, L4, adult	SE	RPMI 1640 medium containing 20% (v/v) sheep serum	Untargeted lipidomic	MSI-2	554 lipids	UHPLC-ESI( +)-MS/MS-Orbitrap	LipidSearch software v.4.1.30 SPI (Thermo Scientific)	(Wang et al., 2018)
		*Nippostrongylus brasiliensis*	Adult	ESPs	RPMI 1640, with 100 U/ml Pen-Strep, 2 mM L-Glutamine and 1% glucose	Targeted metabolomics	MSI-1	2 polar metabolites & 2 lipids	^1^H NMR	Chenomx NMR Suite Professional Software package (v.5.1; Chenomx Inc., Edmonton, Alberta, Canada)	(Nadjsombati et al., 2018)
			L3	SE, ESPs	2% glutamax, 2% pen/strep PBS	Untargeted metabolomics	MSI-1 (polar) MSI-2 (lipids)	164 polar metabolites & 346 lipids	Q-Exactive Orbitrap MS/HPLC	KEGG, MetaCyc, LIPIDMAPS, PubChem CID, HMDB, CTS (https://cts.fiehnlab.ucdavis.edu) databases; open-source software IDEOM; MetaboAnalyst 3.0	(Wangchuk et al., 2019b; Yeshi et al., 2020)
			Adult	ESPs	2% glutamax, 2% pen/strep PBS	Targeted metabolomics	MSI-1	36 polar metabolites & 14 lipids	GC–MS	Agilent’s Mass Hunter Quantitative Analysis software (v.7); MAML; MHL; KEGG	
			Adult	ESPs	RPMI-1640 with 1% antibiotic–antimycotic	Untargeted metabolomics	MSI-1	45 metabolites	UHPLC-MS	XCMS software; MetaboAnalyst, HMDB, and PubChem	(Chen et al., 2021)
				Intestinal content of infected mice	NA			301 metabolites			
		*Oesophagostomum dentatum*; *O. quadrispinulatum*	L3, L4, adult	SE	Basic medium	Untargeted lipidomics	MSI-1	32 lipids	GC	MIDI system (Microbial ID Inc.) software package (MIS version, no. 3.30)	(Joachim et al., 2000)
	Rhabditida	*Ancylostoma caninum*	Adult	SE, ESPs	2% glutamax, 2% pen/strep PBS	Targeted metabolomics	MSI-1	75 polar metabolites & 31 lipids	GC–MS; LC–MS	Agilent’s Mass Hunter Quantitative Analysis software (v.7), MAML; MetaboAnalyst 3.0	(Wangchuk et al., 2019c)
		*Dictyocaulus viviparus*	Eggs, L1-L3, preadult stage, adult	SE	NA	Targeted lipidomic	MSI-1	31 lipids	GC	GLC	(Becker et al., 2017)
		*Necator americanus*	L3	SE, ESPs	2% glutamax, 2% pen/strep PBS	Untargeted metabolomics	MSI-1 (polar) MSI-2 (lipids)	115 polar metabolites & 530 lipids	Q-Exactive Orbitrap MS/HPLC	KEGG, MetaCyc, LIPIDMAPS, PubChem CID, HMDB, CTS databases; open-source software IDEOM; MetaboAnalyst 3.0	(Wangchuk et al., 2021)
		*Strongyloides ratti*	L1, L3, free-living	SE	NA	Targeted lipidomics	MSI-1	12 lipids	GC–MS	Comparing retention times of unknowns with standards	(Minematsu et al., 1990)
Trematoda	Diplostomida	*Schistosoma mansoni*	Adult	SE	RPMI-1640 with pen/strep, glutamine, glucose, NaHCO_3_ and HEPES	Targeted lipidomic	MSI-1	16 lipids	MALDI MSI ( +)	METLIN database	(Ferreira et al., 2014b)
			Eggs, miracidia, cercariae	SE	NA	Untargeted lipidomic	MSI-2	20 lipids	ESI( +)-HRMS	Lipid MAPS & METLIN online databases	(Ferreira et al., 2014a)
			Praziquantel treated adult	SE	NA	Untargeted lipidomic	MSI-2	6 lipids	MALDI-MSI( +)	Lipid MAPS & METLIN databases	(Ferreira et al., 2015)
			Adult	TS	NA	Targeted lipidomic	MSI-2	27 lipids	HPLC–MS (Sciex 4000QTRAP)	Universal HPLC–MS method	(Retra et al., 2015)
			Eggs, cercariae, adult	SE, ESPs	M199 medium with HEPES, antimycotics and L-glutamine	Targeted lipidomic	MSI-2	45 lipids	LC–MS/MS (QTrap) (ESI-)	LipidBlast database; FiehnO lipid database in MS-DIAL (v2.74)	(Giera et al., 2018)
						Targeted lipidomic (total FAs)		28 lipids	GC–MS		
						Targeted lipidomic		276 lipids	LC–MS/MS (QToF) (ESI +)		
			Adult	SE	NA	Untargeted lipidomic	MSI-2	320 lipids	AP-SMALDI MSI	SwissLipids database; LipidMatch (v2.0.2); Lipid Data Analyzer (v2.6.2)	(Kadesch et al., 2020)
Cestoda	Cyclophyllidea	*Dipylidium caninum*	Adult	ESPs	2% glutamax, 2% pen/strep PBS	Targeted metabolomics	MSI-1	35 polar metabolites & 14 lipids	GC–MS	MHL, NIST library, MAML	(Wangchuk et al., 2019a)
		*Echinococcus multilocularis*	Larval metacestode stage	CS	DMEM (10% FBS, 100 U/ml penicillin, 100 μg/ml streptomycin, and 5 μg/ml tetracycline	Untargeted metabolomics	MSI-1	21 polar metabolites	^1^H NMR	Chenomx NMR Suit (v 8.2 with Java 1.8.0_74(× 86)); HMDB; STOCSY with the script IMPACTS (v 1.0.0) in Matlab (V R2015b 8.6.0.267245)	(Ritler et al., 2019)
		*Hymenolepis diminuta*	Infective stage	SE	NA	Targeted lipidomics	MSI-1	62 lipids	TLC, CC, GLC	Chromatography technique	(Ginger & Fairbairn, 1966)

*ESPs* excretory/secretory products; *SE* somatic extract; *SC* Somatic cryosection; *TS* Tegumental surface; *CS* Culture supernatant; *VF* Vesicle fluid; L1, L2, L3 larval stages 1, 2, and 3; *xL3* exsheathed L3. *EVs* extracellular vesicles; ^1^H NMR proton nuclear magnetic resonance; *MS* mass spectrometry; *GC* gas chromatography; *LC* liquid chromatography; *HPLC* high performance liquid chromatography; *TLC* thin layer chromatography; *CC* column chromatography; *GLC* gas liquid chromatography; *UHPLC* ultra high performance liquid chromatography; *Qtrap *quadrupole ion trap; *Q-TOF* quadrupole time-of-flight; *AP-SMALDI MSI* atmospheric pressure (AP) matrix-assisted laser desorption/ionisation (MALDI) mass spectrometry imaging (MSI); *HRMS* high-resolution mass spectrometry; ESI( ±)electrospray ionisation positive/negative mode; *PCA *principle component analysis; *KEGG* Kyoto Encyclopedia of Genes and Genomics; HMDB Human Metabolome Database; *CTS* the Chemical Translation services; *MHL* Mass Hunter Library; *MAML* the in-house Metabolomics Australia metabolite library; *METLIN* metabolite and chemical entity database; *NIST* the national institute of standards and technology; *MetaCyc* metabolic pathways and enzymes database; *Matlab* matrix laboratory; *STOCSY* statistical total correlation spectroscopy; *IDEOM* an Excel interface for analysis of LC–MS-based metabolomics data; *OPLS-DA* Orthogonal Projections to Latent Structures Discriminant Analysis; *NA* not available Modified Ringer-Locke’s solution: 1L of distilled water: 500 mg procaine penicillin, 600 mg streptomycin sulfate, 20 mg mycostatin (Squibb, Nystatin), 9 mg sodium chloride, 0.2 mg sodium bicarbonate, 0.42 mg calcium chloride (the solution was heated at 38 °C; Eagle’s minimum essential medium (GIBCO): glucose 1 g/l (sodium bicarbonate 2.2 g/l and antibiotic/antimycotic 100 × GIBCO 10 ml/l solution)

## Polar metabolites identified from excretory-secretory products and tissue extracts

**Fig. 1:**
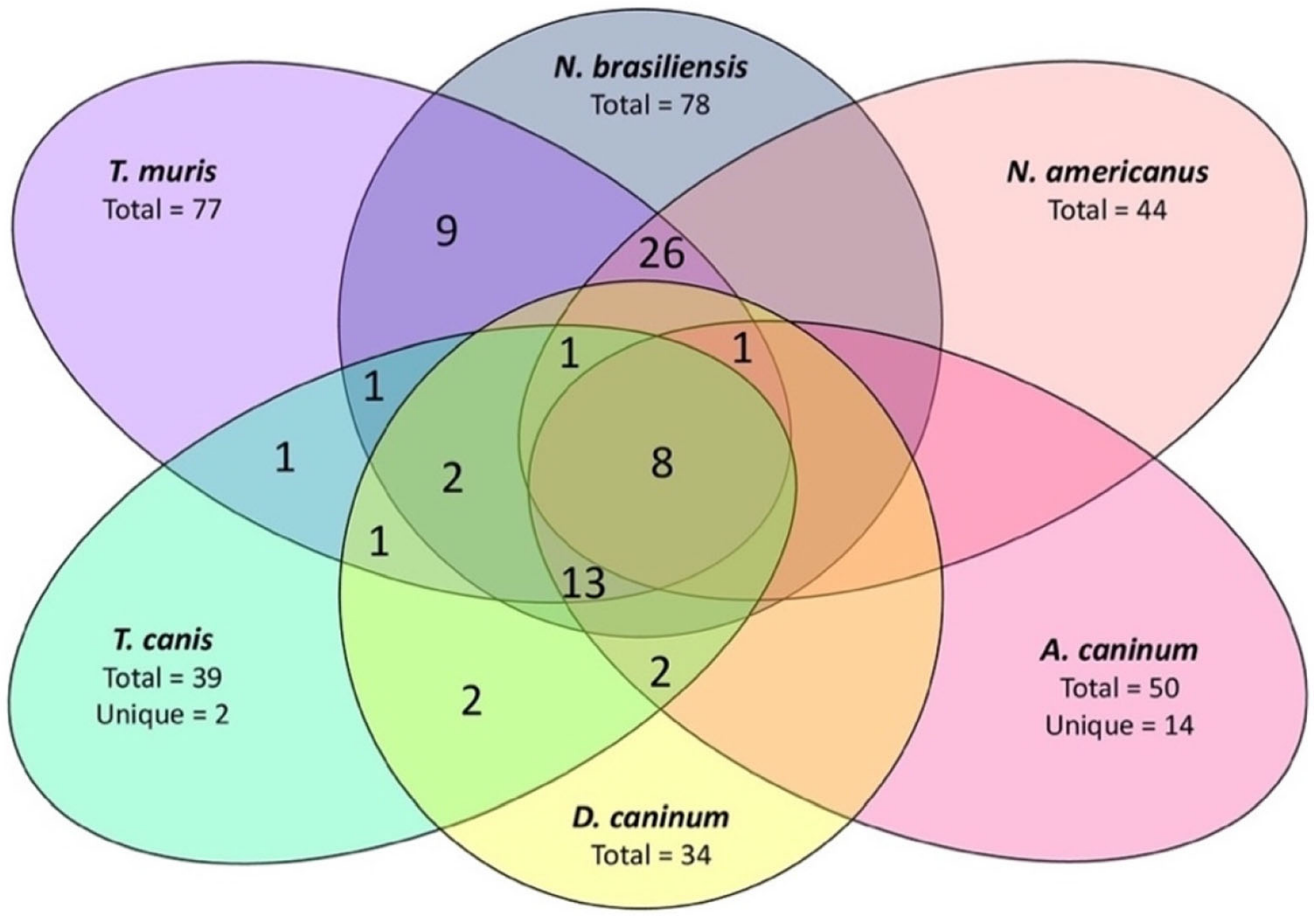
Venn diagram showing both common and uncommon MSI-1 identified polar metabolites among different helminth species

Before the widespread use of NMR spectroscopy, the metabolic profile of body fluids and tissues of hosts infected with parasitic helminths such as *Schistosoma japonicum* and *S. mansoni* was studied (Nishina et al., 1994). However, recent advancements in metabolomics tools and techniques have allowed identifying metabolites produced by helminths under in vitro culture conditions. Through content analysis of the available literature, it was found that a total of 100 polar metabolites (i.e., after excluding common duplicates) were identified and confirmed with available standards (MSI-level-1 identification) from six parasitic helminths (*A. caninum*, *T. canis*, *N. brasiliensis*, *T. muris*, *N. americanus*, and *D. caninum*) (Wangchuk et al., 2019a, 2019b, 2020; Yeshi et al., 2020) (Table 2). The most abundant polar metabolites in the SE were amino acids, carboxylic acids, and derivatives, while ESPs mainly contained sugars and sugar alcohols. Among these six helminths, eight polar metabolites were in common, including D-glucose-6-phosphate, L-alanine, L-methionine, L-phenylalanine, L-tyrosine, mannitol, succinic acid, and 5-oxoproline (Fig. 1; Table 2) (Wangchuk et al., 2019b, 2020; Yeshi et al., 2020). Although bacterial species also secrete succinic acid (Müller et al., 2012), it is more likely that it is a true metabolite of these six parasitic helminths, given that the six helminth studies used 5% antibiotic/antimycotic (A/A) for removing host fecal debris and washing the parasites (3–5 times washes). An additional 2% A/A was used for worm culturing media, which reduces the possibility of microbial contamination (Fig. 1). Moreover, the presence of succinic acid in the ESPs of *N. brasiliensis* was confirmed using ^1^H NMR (Nadjsombati et al., 2018).

**Table 2:** Polar metabolites identified (MSI-1 confirmed) from the excretory-secretory products and tissue extracts of helminths (Fairbairn, 1958a; Nadjsombati et al., 2018; Wangchuk et al., 2019a, 2019b, 2019c, 2020; Yeshi et al., 2020)

Polar metabolites	Helminth and life-cycle stage	Sample types	**Formula**	Chemical sub class*
Adenine	*Nippostrongylus brasiliensis* (L3); *Trichuris muris* (embryonated eggs, adult); *Necator americanus* (L3)	SE, ESPs	C_5_H_5_N_5_	Purines and purine derivatives
Adenosine	*N. brasiliensis* (L3); *T. muris* (embryonated eggs); *N. americanus* (L3)	SE	C_10_H_13_N_5_O_4_	Purine nucleosides
Adenosine 5’-monophosphate	*N. brasiliensis* (L3); *T. muris* (embryonated eggs); *N. americanus* (L3)	SE	C_10_H_14_N_5_O_7_P	Purine ribonucleotides
Aminobutyric acid	*N. brasiliensis* (adult); *T. muris* (adult)	ESPs	C_4_H_9_NO_2_	Amino acids, peptides, and analogues
Asparagine	*Ancylostoma caninum* (adult)	SE, ESPs	C_4_H_8_N_2_O_3_	Amino acids, peptides, and analogues
Azelaic acid	*N. brasiliensis* (L3); *T. muris* (embryonated eggs)	SE, ESPs	C_9_H_16_O_4_	Fatty acids and conjugates
Betaine	N*. brasiliensis* (L3); *T. muris* (embryonated eggs); *N. americanus* (L3)-SE	SE, ESPs	C_5_H_11_NO_2_	Amino acids, peptides, and analogues
Choline	*N. brasiliensis* (L3); *T. muris* (embryonated eggs); *N. americanus* (L3)-SE	SE, ESPs	C_5_H_13_NO	Quarternary ammonium salts
***cis***-Aconitic acid	*N. brasiliensis* (adult); *T. muris* (adult); *Toxocara canis* (adult); *Dipylidium caninum* (adult)	ESP	C_6_H_6_O_6_	Tricarboxylic acids and derivatives
Citric acid	*N. brasiliensis* (adult); *T. muris* (adult); *A. caninum* (adult); *T. canis* (adult); *D. caninum* (adult)	SE, ESPs	C_6_H_8_O_7_	Tricarboxylic acids and derivatives
Deoxyadenosine	*N. brasiliensis* (L3); *T. muris* (embryonated eggs)	SE	C_10_H_13_N_5_O_3_	Purine 2’-deoxyribonucleosides
Glucose 6-phosphate	*N. brasiliensis* (L3, adult); *T. muris* (embryonated eggs, adult); *A. caninum* (adult); *T. canis* (adult*); D. caninum* (adult); *N. americanus (L3)*	SE, ESPs	C_6_H_13_O_9_P	Carbohydrates and carbohydrate conjugates
Glyceric acid	*N. brasiliensis* (L3); *T. muris* (embryonated eggs); *A. caninum* (adult); *T. canis* (adult); *N. americanus* (L3)	SE, ESPs	C_3_H_6_O_4_	Carbohydrates and carbohydrate conjugates
Polar metabolites	Helminth and life-cycle stage	Sample types	Formula	Chemical sub class*
D-Fructose	*T. muris* (adult); *T. canis* (adult); *D. caninum* (adult)	ESPs	C_6_H_12_O_6_	Carbohydrates and carbohydrate conjugates
Fructose-6-phosphate	*T. muris* (adult); *T. canis* (adult)	ESPs	C_6_H_13_O_9_P	Carbohydrates and crbohydrate conjugates
Fumaric acid	*A. caninum* (adult)	SE, ESPs	C_4_H_4_O_4_	Dicarboxylic acids and derivatives
Galactose-6-phosphate	*N. brasiliensis* (adult); *T. muris* (adult); *T. canis* (adult); *D. caninum* (adult)	ESPs	C_6_H_13_O_9_P	Carbohydrates and carbohydrate conjugates
γ-Aminobutyric acid	*A. caninum* (adult); *T. canis* (adult); *D. caninum* (adult)	SE, ESPs	C_4_H_9_NO_2_	Amino acids, peptides, and analogues
Gluconic acid	*N. brasiliensis* (adult); *T. muris* (adult); *A. caninum* (adult); *T. canis* (adult); *D. caninum* (adult)	SE, ESPs	C_6_H_12_O_7_	Carrbohydrates and carbohydrate conjugates
Gluconic-δ-lactone	*T. canis* (adult)	ESPs	C_6_H_10_O_6_	Carbohydrates and carbohydrate conjugates
Glucosamine	*N. brasiliensis* (adult); *T. muris* (adult); *T. canis* (adult)	ESPs	C_6_H_13_NO_5_	Carbohydrates and carbohydrate conjugates
D-Glucose	*N. brasiliensis* (adult); *A. caninum* (adult); *T. canis* (adult); *D. caninum* (adult); *Porrocaecum decipiens* (L4)	SE, ESPs	C_6_H_12_O_6_	Carbohydrates and carbohydrate conjugates
D-Glutamine	*T. canis* (adult); *D. caninum* (adult)	ESPs	C_5_H_10_N_2_O_3_	Amino acids, peptides, and analogues
Glycerol	*N. brasiliensis* (adult); *T. muris* (adult); *A. caninum* (adult); *T. canis* (adult); *D. caninum* (adult)	SE, ESPs	C_3_H_8_O_3_	Carbohydrates and carbohydrate conjugates
**Glycogen**	*P. decipiens* (L4)	SE	C_24_H_42_O_21_	Carbohydrates and carbohydrate conjugates
Glycerol 2-phosphate	*N. brasiliensis* (adult); *T. muris* (adult); *A. caninum* (adult); *T. canis* (adult); *D. caninum* (adult)	SE, ESPs	C_3_H_9_O_6_P	Glycerophosphates
Homogentisate	*N. brasiliensis* (L3); *T. muris* (embryonated eggs)	SE, ESPs	C_8_H_8_O_4_	Phenylacetic acids
L-Homoserine	*A. caninum* (adult)	SE, ESPs	C_4_H_9_NO_3_	Amino acids, peptides, and analogues
Hypoxanthine	*N. brasiliensis* (L3); *T. muris* (embryonated eggs); *N. americanus* (L3)	SE, ESPs	C_5_H_4_N_4_O	Purines and purine derivatives
Inosine	*N. brasiliensis* (L3); *T. muris* (embryonated eggs); *N. americanus* (L3)	SE	C_10_H_12_N_4_O_5_	Purine nucleosides
Polar metabolites	Helminth and life-cycle stage	Sample types	Formula	Chemical sub class*
Isocitrate	*N. brasiliensis* (L3, adult); *T. muris* (embryonated eggs, adult); *D. caninum* (adult); *N. americanus* (L3), *T. canis* (adult)	SE, ESPs	C_6_H_8_O_7_	Tricarboxylic acids and derivatives
L-Isoleucine	A. caninum (adult)	SE, ESPs	C_6_H_13_NO_2_	Amino acids, peptides, and analogues
Lactic acid	*N. brasiliensis* (adult); *T. muris* (adult); *A. caninum* (adult); *T. canis* (adult); *D. caninum* (adult)	SE, ESPs	C_3_H_6_O_3_	Alpha hydroxy acids and derivatives
α-Lactose	*N. brasiliensis* (L3); *T. muris* (embryonated eggs); *N. americanus* (L3)	SE, ESPs	C_12_H_22_O_11_	Carbohydrates and carbohydrate conjugates
L-Alanine	*N. brasiliensis* (L3, adult); *T. muris* (embryonated eggs, adult); *A. caninum* (adult); *T. canis* (adult); *D. caninum* (adult); *N. americanus* (L3)	SE, ESPs	C_3_H_7_NO_2_	Amino acids, peptides, and analogues
L-Arginine	*N. brasiliensis* (L3); *T. muris* (embryonated eggs); *N. americanus* (L3)	SE, ESPs	C_6_H_14_N_4_O_2_	Amino acids, peptides, and analogues
L-Aspartic acid	*N. brasiliensis* (L3, adult); *T. muris* (embryonated eggs, adult); *A. caninum (*adult); *D. caninum* (adult); *N. americanus* (L3)-SE	SE, ESPs	C_4_H_7_NO_4_	Amino acids, peptides, and analogues
L-Carnitine	*N. brasiliensis* (L3); *T. muris* (embryonated eggs); *N. americanus* (L3)-SE	SE, ESPs	C_7_H_15_NO_3_	Quarternary ammonium salts
L-Citrulline	*N. brasiliensis* (L3); *T. muris* (embryonated eggs); *N. americanus* (L3)	SE	C_6_H_13_N_3_O_3_	Amino acids, peptides, and analogues
L-Glutamate	*N. brasiliensis* (L3); *T. muris* (embryonated eggs); *N. americanus* (L3)	SE, ESPs	C_5_H_9_NO_4_	Amino acids, peptides,and analogues
L-Glutamine	*N. brasiliensis* (L3, adult); *T. muris* (embryonated eggs, adult); *N. americanus (*L3)	SE, ESPs	C_5_H_10_N_2_O_3_	Amino acids, peptides, and analogues
Polar metabolites	Helminth and life-cycle stage	Sample types	Formula	Chemical sub class*
L-Histidine	*N. brasiliensis* (L3); *T. muris* (embryonated eggs); *A. caninum* (adult); *N. americanus* (L3)	SE, ESPs	C_6_H_9_N_3_O_2_	Amino acids, peptides, and analogues
L-Leucine	*N. brasiliensis* (L3); *T. muris* (embryonated eggs); *N. americanus* (L3)-SE	SE, ESPs	C_6_H_13_NO_2_	Amino acids, peptides, and analogus
L-Lysine	*N. brasiliensis* (L3); *T. muris* (embryonated eggs); *A. caninum* (adult); *N. americanus* (L3)	SE, ESPs	C_6_H_14_N_2_O_2_	Amino acids, peptides, and analogues
L-Methionine	*N. brasiliensis* (L3, adult); *T. muris* (embryonated eggs, adult); *A. caninum* (adult); *T. canis* (adult); *D. caninum* (adult); *N. americanus* (L3)	SE, ESPs	C_5_H_11_NO_2_S	Amino acids, peptides, and analogues
L-Phenylalanine	*N. brasiliensis* (L3, adult); *T. muris* (embryonated eggs, adult); *A.* caninum (adult); *T. canis* (adult); *D. caninum* (adult); *N. americanus* (L3)	SE, ESPs	C_9_H_11_NO_2_	Amino acids, peptides, and analogues
L-Pipecolic acid	*N. brasiliensis* (L3); *T. muris* (embryonated eggs); *N. americanus* (L3)	SE, ESPs	C_6_H_11_NO_2_	Amino acids, peptides, and analogues
L-Proline	*N. brasiliensis* (L3); *T. muris* (embryonated eggs); *A. caninum* (adult); *N. americanus* (L3)	SE, ESPs	C_5_H_9_NO_2_	Amino acids, peptides, and analogues
L-Serine	*N. brasiliensis* (L3); *T. muris* (embryonated eggs); *A. caninum* (adult); *N. americanus* (L3)	SE, ESPs	C_3_H_7_NO_3_	Amino acids, peptides, and analogues
L-Threonine	*N. brasiliensis* (L3); *T. muris* (embryonated eggs); *A. caninum* (adult); *N. americanus* (L3)	SE	C_4_H_9_NO_3_	Amino acids, peptides, and analogues
L-Tryptophan	*N. brasiliensis* (L3, adult); *T. muris* (embryonated eggs, adult); *A. caninum* (adult); *T. canis* (adult); *N. americanus* (L3)	SE, ESPs	C_11_H_12_N_2_O_2_	Indolyl carboxylic acids and derivatives
Polar metabolites	Helminth and life-cycle stage	Sample types	Formula	Chemical sub class*
L-Tyrosine	*N. brasiliensis* (L3, adult); T*. muris* (embryonated eggs, adult); *A. caninum* (adult); *T. canis* (adult); *D. caninum* (adult); *N. americanus* (L3)	SE, ESPs	C_9_H_11_NO_3_	Amino acids, peptides, and analogues
L-Valine	*N. brasiliensis* (L3); *T. muris* (embryonated eggs); *A. caninum* (adult); *N. americanus* (L3)	SE, ESPs	C_5_H_11_NO_2_	Amino acids, peptides, and analogues
2-Aminoadipic acid	*N. brasiliensis* (L3); *T. muris* (embryonated eggs); *N. americanus* (L3)	SE, ESPs	C_6_H_11_NO_4_	Amino acids, peptides, and analogues
(2S,6S)-2,6-Diaminoheptanedioic acid	*N. brasiliensis* (L3)	ESPs	C_7_H_14_N_2_O_4_	Amino acids, peptides, and analogues
Maleic acid	*N. brasiliensis* (L3); *T. muris* (embryonated eggs)	SE	C_4_H_4_O_4_	Dicarboxylic acids and derivatives
Malic acid	*N. brasiliensis* (adult); *T. muris* (adult); *A. caninum* (adult); *T. canis* (adult); *D. caninum* (adult)	SE, ESPs	C_4_H_6_O_5_	Beta hydroxy acids and derivatives
D-Maltose	*N. brasiliensis* (adult); *T. muris* (adult); *A. caninum* (adult); *T. canis* (adult); *D. caninum (*adult)	SE, ESPs	C_12_H_22_O_11_	Carbohydrates and carbohydrate conjugates
Mannitol	*N. brasiliensis* (L3, adult); *T. muris* (embryonated eggs); *A. caninum* (adult); *T. canis* (adult); *D. caninum* (adult); *N. americanus* (L3)	SE, ESPs	C_6_H_14_O_6_	Carbohydrates and carbohydrate conjugates
Erythritol	*N. brasiliensis* (adult); *T. muris* (adult); *A. caninum* (adult); *T. canis* (adult); *D. caninum* (adult)	SE, ESPs	C_4_H_10_O_4_	Carbohydrates and carbohydrate conjugates
*myo*-Inositol	*N. brasiliensis* (adult); *T. muris* (adult); *A. caninum* (adult); *T. canis* (adult); *D. caninum* (adult)	SE, ESPs	C_6_H_12_O_6_	Alcohols and polyols
*myo*-Inositol 1-phosphate	*A. caninum* (adult)	SE, ESPs	C_6_H_13_O_9_P	Alcohols and polyols
N-Acetylputrescine	*N. brasiliensis* (L3); *T. muris* (embryonated eggs); *N. americanus* (L3)	SE, ESPs	C_6_H_14_N_2_O	Carboximidic acids
Polar metabolites	Helminth and life-cycle stage	Sample types	Formula	Chemical sub class*
3-Methylhistidine	*N. brasiliensis* (L3); *T. muris (*embryonated eggs)	SE, ESPs	C_7_H_11_N_3_O_2_	Amino acids, peptides, and analogues
N6,N6,N6-Trimethyl-L-lysine	*N. brasiliensis* (L3); *T. muris* (embryonated eggs); *N. americanus* (L3)	SE, ESPs	C_9_H_20_N_2_O_2_	Amino acids, peptides, and analogues
Ornithine	*A. caninum* (adult)	SE, ESPs	C_5_H_12_N_2_O_2_	Amino acids, peptides, and analogues
Orotatic acid	*N. brasiliensis* (L3)	ESPs	C_5_H_4_N_2_O_4_	Pyrimidines and pyrimidine derivatives
Oxalic acid	*A. caninum* (adult)	SE, ESPs	C_2_H_2_O_4_	Dicarboxylic acids and derivatives
Phosphoenolpyruvic acid	*N. brasiliensis* (adult); *T. muris* (adult); *A. caninum* (adult); *T. canis* (adult); *D. caninum* (adult)	SE, ESPs	C_3_H_5_O_6_P	Phosphate esters
Pterin	*N. brasiliensis* (L3)	ESPs	C_6_H_5_N_5_O	Pterins and derivatives
Pyridoxal	*N. brasiliensis* (L3); *T. muris* (embryonated eggs); *N. americanus* (L3)	SE	C_8_H_9_NO_3_	Pyridine and derivatives
*p*-Hydroxyphenylacetic acid	*N. brasiliensis* (adult); *T. muris* (adult)	ESPs	C_8_H_8_O_3_	1-Hydroxy-2-unsubstituted benzenoids
Rhamnose	*A. caninum* (adult)	SE, ESPs	C_6_H_12_O_5_	Carbohydrates and carbohydrate conjugates
Ribitol	*N. brasiliensis* (adult); *T. muris* (adult); *T. canis* (adult); *D. caninum* (adult)	ESPs	C_5_H_12_O_5_	Carbohydrates and carbohydrate conjugates
D-Ribose	*A. caninum* (adult)	SE, ESPs	C_5_H_10_O_5_	Carbohydrates and carbohydrate conjugates
Scyllo-inositol	*T. canis (*adult); *D. caninum* (adult)	ESPs	C_6_H_12_O_6_	Alcohols and polyols
Sorbitol	*N. brasiliensis* (adult); *A. caninum* (adult); *T. canis* (adult); *D. caninum* (adult)	SE, ESPs	C_6_H_14_O_6_	Carbohydrates and carbohydrate conjugates
Succinic acid	*N. brasiliensis* (L3, adult); *T. muris* (embryonated eggs, adult); *A. caninum* (adult); *T. canis* (adult); *D. caninum* (adult); *N. americanus* (L3)	SE, ESPs	C_4_H_6_O_4_	Dicarboxylic acids and derivatives
Sucrose	*A. caninum* (adult); *T. canis* (adult); *D. caninum* (adult)	SE, ESPs	C_12_H_22_O_11_	Carbohydrates and carbohydrate conjugates
Polar metabolites	Helminth and life-cycle stage	Sample types	Formula	Chemical sub class*
Malic acid	*N. brasiliensis* (L3); *T. muris* (embryonated eggs); *N. americanus* (L3)-SE	SE, ESPs	C_4_H_6_O_5_	Beat hydroxy acids and derivatives
Talose	*N. brasiliensis* (adult); *A. caninum* (adult); *T. canis* (adult); *D. caninum* (adult)	SE, ESPs	C_6_H_12_O_6_	Carbohydrates and carbohydrate conjugates
Tartaric acid	*N. brasiliensis* (adult); *T. muris* (adult); *A. caninum* (adult); *T. canis* (adult); *D. caninum* (adult)	SE, ESPs	C_4_H_6_O_6_	Carbohydrates and carbohydrate conjugates
Thymine	*N. brasiliensis* (L3); *T. muris* (embryonated eggs)	SE	C_5_H_6_N_2_O_2_	Pyrimidines and pyrimidine derivatives
4-Hydroxyproline	*N. brasiliensis* (adult); *T. muris* (adult); *A. caninum* (adult); *T. canis* (adult); *D. caninum* (adult)	SE, ESPs	C_5_H_9_NO_3_	Amino acids, peptides, and analogues
Trehalose	*A. caninum* (adult); *A. lumbricoides* (eggs); *Porrocaecum decipiens* (L4)	SE, ESPs	C_12_H_22_O_11_	Carbohydrates and carbohydrate conjugates
Turanose	*A. caninum* (adult)	SE, ESPs	C_12_H_22_O_11_	Fatty acyl glycosides
Urea	*A. caninum* (adult)	SE, ESPs	CH_4_N_2_O	Ureas
Uridine	*T. muris* (adult); *A. caninum* (adult)	SE, ESPs	C_9_H_12_N_2_O_6_	Pyrimidine nucleosides
Urocanic acid	*N. brasiliensis* (L3); *T. muris* (embryonated eggs); *N. americanus* (L3)	SE, ESPs	C_6_H_6_N_2_O_2_	Imidazoles
Xanthine	*N. brasiliensis* (L3); *T. muris* (embryonated eggs); *N. americanus* (L3)	SE, ESPs	C_5_H_4_N_4_O_2_	Purines and purine derivatives
D-Xylose	*N. brasiliensis* (adult); *T. muris* (adult); *A. caninum* (adult); *T. canis* (adult); *D. caninum* (adult)	SE, ESPs	C_5_H_10_O_5_	Carbohydrates and carbohydrate conjugates
5-Aminolevulinic acid	*N. brasiliensis* (L3); *T. muris* (embryonated eggs); *N. americanus* (L3)	SE	C_5_H_9_NO_3_	Amino acids, peptides, and analogues
4-Hydroxybenzoate	*N. brasiliensis* (L3); *T. muris* (embryonated eggs); *N. americanus* (L3)	SE, ESPs	C_7_H_6_O_3_	Benzoic acids and derivatives
Polar metabolites	Helminth and life-cycle stage	Sample types	Formula	Chemical sub class*
Oxoglutaric acid	*N. brasiliensis* (L3); *T. muris* (embryonated eggs); *N. americanus* (L3)	SE, ESPs	C_5_H_6_O_5_	Gamma keto acids and derivatives
Cyclic AMP	*N. brasiliensis* (L3); *T. muris* (embryonated eggs); *N. americanus* (L3)	SE, ESPs	C_10_H_12_N_5_O_6_P	Cyclic purine nucleotides
Pyroglutamic acid	*N. brasiliensis* (L3, adult); *T. muris* (adult); *A. caninum* (adult); *T. canis* (adult); *D. caninum* (adult); *N. americanus* (L3)	ESPs	C_5_H_7_NO_3_	Amino acids, peptides, and analogues
Gentisic acid	*N. brasiliensis* (L3)	ESPs	C_7_H_6_O_4_	Benzoic acids and derivatives
3-phosphoglyceric acid	*A. caninum* (adult); *T. canis* (adult)	SE, ESPs	C_3_H_7_O_7_P	Carbohydrates and carbohydrate derivatives
4-Trimethylammoniobutanoic acid	*N. brasiliensis* (L3); *T. muris* (embryonated eggs)	SE	C_7_H_15_NO_2_	Fatty acids and conjugates
4-hydroxyphenyl acetate	*N. brasiliensis* (adult); *T. muris* (adult); *A. caninum* (adult)	SE, ESPs	C_8_H_8_O_3_	Phenol esters

*MSI-1 *Metabolomics Standards Initiative Level-1; *SE *somatic extract; *ESPs* excretory/secretory products; *L3*third-stage larvae

*Chemical sub class as described in HMDB and PubChem

Pterin, orotate, LL-2,6-diaminoheptanedioate, and 2,5-dihydroxybenzoate have been identified as polar metabolites unique to infective L3 stage of *N. brasiliensis* in a study by Yeshi et al. (2020). Pterin, a pyrazino-pyrimidine derivative, was first discovered as a fluorescent pigments in butterfly wings by Hopkins in 1894 (Hopkins, 1894) and has since been reported in various living organisms such as cyanobacteria, mammals, and parasites. In humans, monocytes or macrophages produce excess neopterin upon stimulation with IFN-γ (Weiss et al., 1993). Biopterin, a pterin derivative, has been reported as a ROS scavenger (Shen & Zhang, 1993) and was detected in the muscle extract of *A. lumbricoides* through paper chromatographic analysis followed by purification using a neutral pH Ecteola (Epichlorohydrin triethanolamine)-cellulose column (1.8 × 30 cm) and a Sephadex column (G-25, fine, 1.8 × 20 cm) (Fukushima, 1970). However, the specific role of biopterin has not been reported in any available literature.

Orotate, detected in the ESPs of *N. brasiliensis* L3 stage might be the end-product of de novo pyrimidine biosynthesis. Surprising, orotate was not detected in embryonated eggs and adult *T. muris*, despite all five enzymes involved in pyrimidine metabolism being present in helminths, including *N. brasiliensis* and *T. muris* (Yeshi et al., 2020). The role of orotate in living organisms is described as a regulator of genes involved in developing cells, tissues, and organisms as a whole (Loffler et al., 2016). In the somatic tissue extract of *A. caninum*, a non-reducing disaccharide called trehalose (also known as mycose or tremalose) was reported to be present. Trehalose was also detected (< 10% of dry weight) in *A. lumbricoides* eggs (Fairbairn & Passey, 1957) as well as matured larvae of *A. lumbricoides* and *Porrocaecum decipiens* (Kalf & Rieder, 1958). Fairbairn (Fairbairn, 1958b) detected trehalose (a source of energy and carbon) in the somatic tissues of 14 helminth species (refer Table 1 above) and suggested that parasitic nematodes possess more trehalose than trematodes and cestodes. Trehalose is commonly found in yeast and fungi (Elbein et al., 2003).

**Fig. 2:**
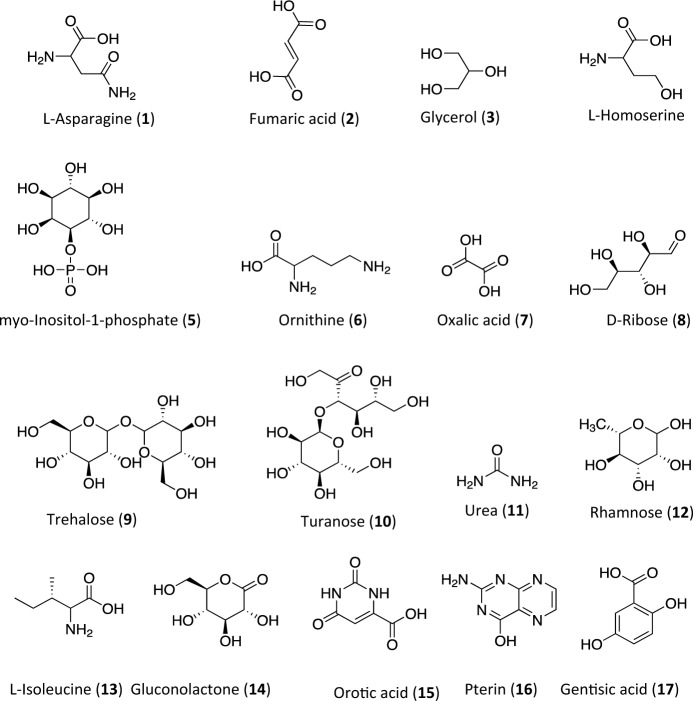
Chemical structures of commonly reported compounds (polar) unique to specific helminth species. Compounds 1–13: unique to adult *A. caninum*; compound 14: unique to adult *T. canis*; compounds 15–17: unique to infective larval third stage (L3) of *N. brasiliensis*

Several metabolites unique to different parasitic helminths have been identified, including amino acids and carboxylic acids, with *A. caninum* (adult) having 13, *N. brasiliensis* infective stage (L3) having three, and adult *T. canis* having one (Fig. 1, Table 1). Additionally, gluconolactone (gluconic-δ-lactone) has been reported as a unique metabolite in the ESPs of adult *T. canis* (Wangchuk et al., 2020). A representative structure of metabolites unique to different parasitic helminths is given in Fig. 2. The presence of species-specific metabolites suggests the potential for developing diagnostic biomarkers. However, the reliability of unique metabolites, both polar metabolites and lipids, may be limited as experimental conditions and analytical platforms differ across studies, and comparisons may not provide a comprehensive picture of the samples under investigation. It is also important to note that the unique or different molecules in the metabolite profiles among the helminths could be due to different experimental conditions, analytical platforms, helminth species, and their different life-cycle stages, which are known to produce stage-specific metabolites (Barrett, 1987; Wangchuk et al., 2019b).

## Lipids of the excretory-secretory products and tissue extracts of helminths

**Fig. 3:**
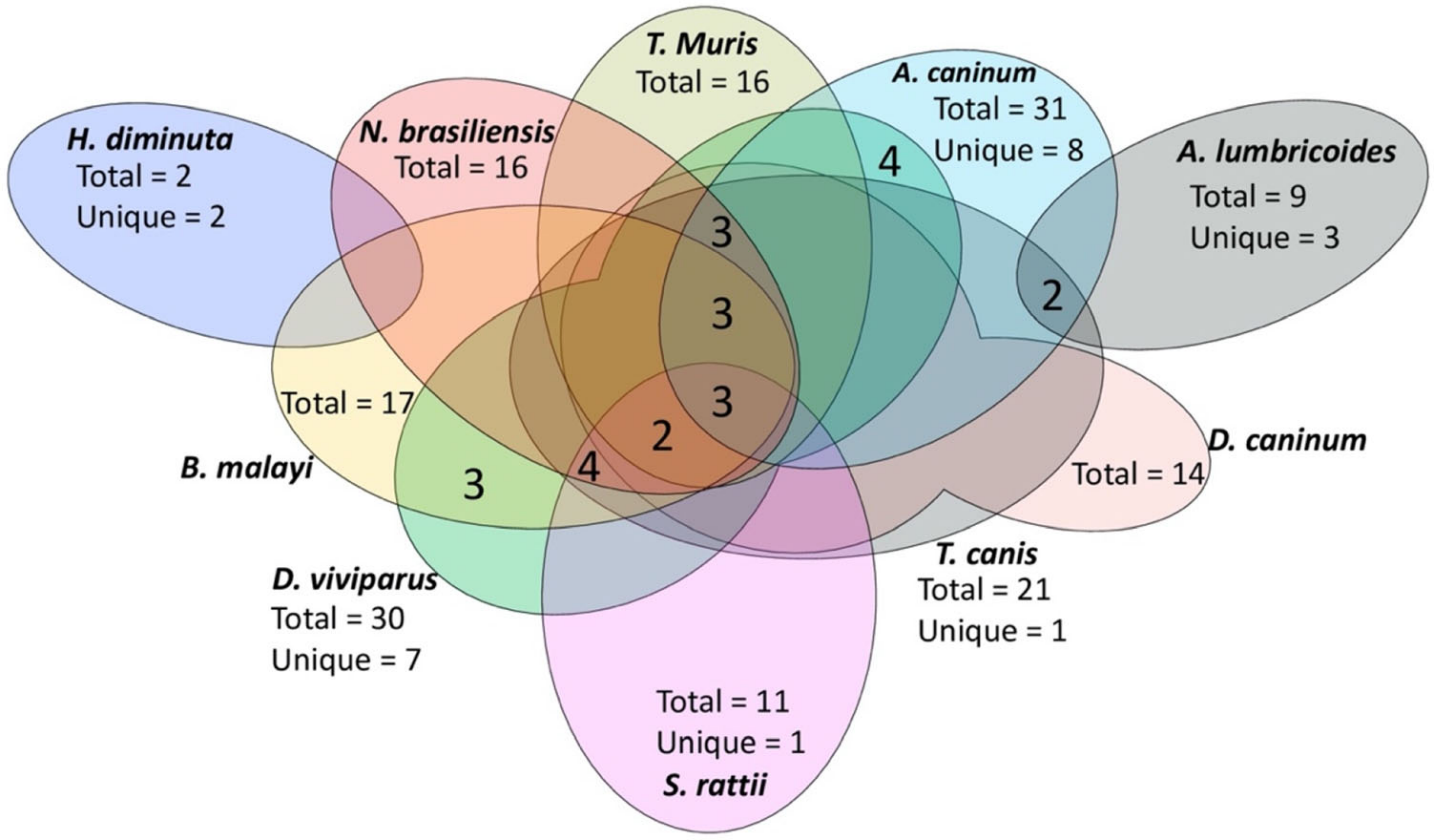
Venn diagrams showing the MSI-1 identified lipids among different helminth species

Out of the 28 helminth metabolomics studies we reviewed, 17 solely focused on lipidomics analysis (as shown in Table 2), using untargeted (8 studies) and targeted (9 studies) approaches. However, only seven of these studies achieved MSI level-1 identification (i.e., confirmed the identity of lipids with authentic standards) (refer to Table 3 and Fig. 3), while 10 studies reported MSI level-2 (putatively identified) lipids. Due to the large number of putative lipids identified, it is not feasible to include them all in this review, and they can be accessed from the references in Table 2. For example, in the infective stages of *N. brasiliensis* and *T. muris*, 350 putative lipids were identified, with glycerophospholipids and glycerolipids being the predominant lipid groups (Yeshi et al., 2020). In *S. mansoni*, Ferreira et al. (2014a) reported the presence of phospholipids and triacylglycerols, with phosphatidylcholines (PCs) being the major lipids (Ferreira et al., 2015). Similarly, Wang et al. (2020) putatively identified 587 lipids from the somatic tissue extract of *Ascaris suum*.

**Table 3:** Lipids identified (MSI-1) from the excretory-secretory products, and tissue extracts of helminths (Greichus & Greichus, 1966; Learmonth et al., 1987; Minematsu et al., 1990; Nadjsombati et al., 2018; Wangchuk et al., 2019a, 2019b, 2019c, 2020; Yeshi et al., 2020)

Lipids	Helminth and life cycle stage	Sample types	Formula	Chemical sub class*
Acetic acid	*Ancylostoma caninum* (adult), *Nippostrongylus brasiliensis* (adult), *Trichuris muris* (adult), *Toxocara canis* (adult), *Ascaris lumbricoides* (adult)	ESPs, SE	C_2_H_4_O_2_	Carboxylic acids
Adrenic acid	*A. caninum* (adult)	ESPs	C_22_H_36_O_2_	Fatty acids and conjugates
Arachidic acid	*Dipylidium caninum* (adult), *N. brasiliensis* (adult), *T. muris* (adult), *T. canis* (adult), *Brugia malayi* (adult), *Dictyocaulus viviparus* (eggs, L1-L3, pre-adult, adult), *A. caninum* (adult)	ESPs, SE	C_20_H_40_O_2_	Fatty acids and conjugates
Arachidonic acid	*D. caninum* (adult), *N. brasiliensis* (adult), *T. muris* (adult), *T. canis* (adult); *Strongyloides rattii* (L1, L3, adult), *B. malayi* (adult), *D. viviparus* (eggs, L1-L3, pre-adult, adult)	ESPs, SE	C_20_H_32_O_2_	Fatty acids and conjugates
Behenic acid	*A. caninum* (adult), *B. malayi* (adult)	ESPs, SE	C_22_H_44_O_2_	Fatty acids and conjugates
Butyric acid	*A. caninum* (adult), *N. brasiliensis* (adult), *T. muris* (adult), *T. canis* (adult), *A. lumbricoides* (adult)	ESPs, SE	C_4_H_7_O_2_	Fatty acids and conjugates
Capric acid	*D. caninum* (adult), *N. brasiliensis* (adult), *T. muris* (adult), *T. canis* (adult), *A. caninum* (adult); *D. viviparus* (eggs, L1-L3, pre-adult, adult)	ESPs	C_10_H_20_O_2_	Fatty acids and conjugates
Caproic acid	*A. lumbricoides* (adult)	ESPs	C_6_H_12_O_2_	Fatty acids and conjugates
Caprylic acid	*A. caninum* (adult), *D. viviparus* (eggs, L1-L3, pre-adult, adult)	ESPs, SE	C_8_H_16_O_2_	Fatty acids and conjugates
Cholesterol	*Hymenolepis diminuta* (infective stage)	SE	C_27_H_46_O	Cholestane steroids
dihomo-γ-linolenic acid	*S. rattii* (L1, L3, adult), *D. viviparus* (eggs, L1-L3, pre-adult, adult), *B. malayi* (adult)	SE	C_20_H_34_O_2_	Fatty acids and conjugates
Docosahexaenoic acid	*N. brasiliensis* (adult), *T. muris* (adult), *D. caninum* (adult), *T. canis* (adult)	ESPs	C_22_H_32_O_2_	Fatty acids and conjugates
Lipids	Helminth and life cycle stage	Sample types	Formula	Chemical sub class*
Eicosadienoic acid	*S. rattii* (L1, L3, adult), *B. malayi* (adult), *D. viviparus* (eggs, L1-L3, pre-adult, adult)	SE	C_20_H_36_O_2_	Fatty acids and conjugates
Eicosapentaenoic acid	*S. rattii* (L1, L3, adult), *D. viviparus* (eggs, L1-L3, pre-adult, adult)	SE	C_20_H_30_O_2_	Fatty acids and conjugates
Elaidic acid	*A. caninum* (adult)	ESPs	C_18_H_34_O_2_	Fatty acids and conjugates
Erucic acid	*A. caninum* (adult)	ESPs	C_22_H_42_O_2_	Fatty acids and conjugates
9Z-Eicosenoic acid	*B. malayi* (adult), *D. viviparus* (eggs, L1-L3, pre-adult, adult)	SE	C_20_H_38_O_2_	Fatty acids and conjugates
Heneicosanoic acid	*A. caninum* (adult)	ESPs	C_21_H_42_O_2_	Fatty acids and conjugates
Isobutyric acid	*A. caninum* (adult), *T. canis* (adult), *A. lumbricoides* (adult)	ESPs, SE	C_4_H_7_O_2_	Carboxylic acids
Isovaleric acid	*A. caninum* (adult), *T. canis* (adult)	ESPs	C_5_H_9_O_2_	Fatty acids and conjugates
Dodecanoic acid	*D. caninum* (adult), *N. brasiliensis* (adult), *T. muris* (adult), *A. caninum* (adult), *T. canis* (adult), *B. malayi* (adult), *D. viviparus* (eggs, L1-L3, pre-adult, adult)	ESPs, SE	C_12_H_24_O_2_	Fatty acids and conjugates
Lecithin	*H. diminuta* (infective stage)	SE	C_44_H_88_NO_8_P	Glycerophosphocholines
Tetracosanoic acid	*A. caninum* (adult)	ESPs	C_24_H_48_O_2_	Fatty acids and conjugates
Linoleic acid	*D. caninum* (adult), *T. canis* (adult), *N. brasiliensis* (adult), *T. muris* (adult), *A. caninum* (adult), *S. rattii* (L1, L3, adult), *D. viviparus* (eggs, L1-L3, pre-adult, adult), *B. malayi* (adult)	ESPs, SE	C_18_H_32_O_2_	Linoleic acids and derivatives
*cis*-Linoleic acid	*D. viviparus* (eggs, L1-L3, pre-adult, adult)	SE	C_17_H_30_O_2_	Linoleic acids and derivatives
*trans*-Linoleic acid	*D. viviparus* (eggs, L1-L3, pre-adult, adult)	SE	C_17_H_30_O_2_	Linoleic acids and derivatives
α-Linolenic acid	*D. viviparus* (eggs, L1-L3, pre-adult, adult)	SE	C_18_H_30_O_2_	Lineolic acids and derivatives
Heptadecanoic acid	*D. caninum* (adult), *T. canis* (adult), *A. caninum* (adult), *N. brasiliensis* (adult), *T. muris* (adult), *D. viviparus* (eggs, L1-L3, pre-adult, adult)	ESPs	C_17_H_34_O_2_	Fatty acids and conjugates
Lipids	Helminth and life cycle stage	Sample types	Formula	Chemical sub class*
Myristic acid	*D. caninum* (adult), *N. brasiliensis* (adult), *T. muris* (adult), *T. canis* (adult), *A. caninum* (adult), *B. malayi* (adult), *D. viviparus* (eggs, L1-L3, pre-adult, adult)	ESPs, SE	C_14_H_28_O_2_	Fatty acids and conjugates
Myristic acid	*D. caninum* (adult), *N. brasiliensis* (adult), *T. muris* (adult), *T. canis* (adult), *A. caninum* (adult), *B. malayi* (adult), *D. viviparus* (eggs, L1-L3, pre-adult, adult)	ESPs, SE	C_14_H_28_O_2_	Fatty acids and conjugates
Myristoleic acid	*B. malayi* (adult), *D. viviparus* (eggs, L1-L3, pre-adult, adult)	SE	C_14_H_26_O_2_	Fatty acids and conjugates
Nervonic acid	*A. caninum* (adult), *D. viviparus* (eggs, L1-L3, pre-adult, adult)	ESPs	C_24_H_46_O_2_	Fatty acids and conjugates
Nonadecanoic acid	*A. caninum* (adult)	ESPs	C_19_H_38_O_2_	Fatty acids and conjugates
Oleic acid	*N. brasiliensis* (adult), *T. muris* (adult), *S. rattii (*L1, L3, adult), *B. malayi* (adult), *D. caninum* (adult), *T. canis* (adult), *D. viviparus* (eggs, L1-L3, pre-adult, adult)	ESPs, SE	C_18_H_34_O_2_	Fatty acids and conjugates
Palmitic acid	*D. caninum* (adult), *N. brasiliensis* (adult), *T. muris* (adult), *T. canis* (adult), *A. caninum* (adult), *S. rattii* (L1, L3, adult), *B. malayi* (adult), *D. viviparus* (eggs, L1-L3, pre-adult, adult)	ESPs, SE	C_16_H_32_O_2_	Fatty acids and conjugates
Palmitoleic acid	*S. rattii* (L1, L3, adult), *B. malayi* (adult), *D. viviparus* (eggs, L1-L3, pre-adult, adult)	SE	C_16_H_30_O_2_	Fatty acids and conjugates
Pelargonic acid	*A. caninum* (adult)	ESPs	C_9_H_18_O_2_	Fatty acids and conjugates
Pentadecanoic acid	*D. caninum* (adult), *N. brasiliensis* (adult), *T. muris* (adult), *T. canis* (adult), *A. caninum* (adult), *D. viviparus (*eggs, L1-L3, pre-adult, adult)	ESPs	C_15_H_30_O_2_	Fatty acids and conjugates
Petroselinic acid	*A. caninum* (adult), *D. viviparus* (eggs, L1-L3, pre-adult, adult)	ESPs, SE	C_18_H_34_O_2_	Fatty acids and conjugates
Propionic acid	*A. caninum* (adult), *N. brasiliensis* (adult), *T. muris* (adult), *T. canis* (adult), *A. lumbricoides* (adult)	ESPs, SE	C_3_H_6_O_2_	Carboxylic acids
Lipids	Helminth and life cycle stage	Sample types	Formula	Chemical sub class*
Stearic acid	*D. caninum* (adult), *T. canis* (adult), *A. caninum* (adult), *N. brasiliensis* (adult), *T. muris* (adult), *S. rattii* (L1, L3, adult), *B. malayi* (adult), *D. viviparus* (eggs, L1-L3, pre-adult, adult)	ESPs	C_18_H_36_O_2_	Fatty acids and conjugates
Stearidonic acid	*A. caninum* (adult)	ESPs	C_18_H_28_O_2_	Lineolic acids and derivatives
Tiglic acid	*A. lumbricoides* (adult)	ESPs	C_5_H_8_O_2_	Fatty acids and conjugates
Tridecanoic acid	*D. caninum* (adult), *T. canis* (adult), *N. brasiliensis* (adult), *T. muris* (adult), *A. caninum* (adult)	ESPs	C_13_H_26_O_2_	Fatty acids and conjugates
Tricosanoic acid	*A. caninum* (adult), *D. viviparus* (eggs, L1-L3, pre-adult, adult)	ESPs	C_23_H_46_O_2_	Fatty acids and conjugates
Undecanoic acid	*D. caninum* (adult), *T. canis* (adult), *A. caninum* (adult), *D. viviparus* (eggs, L1-L3, pre-adult, adult)	ESPs	C_11_H_22_O_2_	Fatty acids and conjugates
Valeric acid	*A. lumbricoides* (adult)	ESPs	C_5_H_9_O_2_^−^	Fatty acids and conjugates
α-Linolenic acid	*B. malayi* (adult), *D. viviparus* (eggs, L1-L3, pre-adult, adult), *S. rattii* (L1, L3, adult)	SE	C_18_H_30_O_2_	Lineolic acids and derivatives
α-Glycerophosphorylcholine	*T. canis* (adult)	SE	C_8_H_20_NO_6_P	Glycerophosphocholines
γ-Linolenic acid	*S. rattii* (L1, L3, adult), *B. malayi* (adult)	SE	C_18_H_30_O_2_	Lineolic acids and derivatives
Ethylmethylacetic acid	*A. caninum* (adult), *T. canis* (adult), *A. lumbricoides* (adult)	ESPs	C_5_H_9_O_2_	Fatty acids and conjugates
( ±)-2-Methylpentanoic acid	*A. caninum* (adult), *A. lumbricoides* (adult)	ESPs	C_6_H_12_O_2_	Fatty acids and conjugates
2,5-Dimethyl-2E-tridecenoic acid	*D. viviparus* (eggs, L1-L3, pre-adult, adult)	SE	C_15_H_28_O_2_	Fatty acids and conjugates
7-Methyl-6E-hexadecenoic acid	*D. viviparus* (eggs, L1-L3, pre-adult, adult)	SE	C_17_H_32_O_2_	Fatty acids and conjugates
7,7-Dimethyl-5Z,8Z-eicosadienoic acid	*D. viviparus* (eggs, L1-L3, pre-adult, adult)	SE	C_22_H_40_O_2_	Fatty acids and conjugates

*MSI-1* Metabolomics Standards Initiative level-1; *SE* somatic extract; *ESPs* excretory-secretory products; *L3* third stage larva; *L4* fourth stage larva

*Chemical sub class as described in HMDB, LipidMaps, and PubChem

The identities of 55 lipids produced by 10 different helminth species at various life cycle stages were confirmed through MSI level-1 identification protocols after excluding duplicates (Table 3). Many unique MSI level-1 identified lipids were reported, such as seven unique lipids (elaidic acid, erucic acid, heneicosylic acid, lignoceric acid, nonadecylic acid, pelargonic acid, and stearic acid) from *A. caninum,* five (linoleic acid, linolenic acid, 2,5-dimethyl-2E-tridecenoic acid, 7-methyl-6E-hexadecenoic acid, and 7,7-dimethyl-5Z,8Z-eicosadienoic acid) from *D. viviparus*, three (caproic acid, valeric acid, and tiglic acid) from *A. lumbricoides*, and one in *T. canis* (α-glycerophosphorylcholine) (Table 3). Only three lipids were isolated and identified using ^1^H NMR spectroscopy (i.e., from somatic tissues of *T. canis*) (see Table 2). There were 41 lipids common to all 17 helminths included in Table 3, such as palmitic acid, oleic acid, and stearic acid, which were present in eight helminth species (*A. caninum*, *T. canis*, *D. caninum*, *N. brasiliensis*, *T. muris*, *D. viviparus*, and *S. ratti*).

The presence of fatty acids such as cis-octadecenoic acid, branched-chain, and monoenoic acids like oleic and vaccenic acid can modify the physical properties of host cell membranes and result in cell rupture (Ward, 1982). However, caution should be exercised when considering common and unique lipids among these helminths, as each study's experimental conditions and analytical platforms vary. Stearic acid (C18) was found to be one of the major fatty acids in the ESPs of adult *A. caninum* (Wangchuk et al., 2019c), *T. muris*, and *N. brasiliensis* (Wangchuk et al., 2019b), *T. canis* (Wangchuk et al., 2020), *D. caninum* (Wangchuk et al., 2019a), and *Ascaridia galli* (Ghosh et al., 2010). Additionally, stearic, palmitic, palmitoleic, and oleic acids were the most prevalent fatty acids identified in the free-living (L1 – L3) and parasitic stages of *D. viviparus* (Becker et al., 2017).

Barrett (1981) reported that helminths have a higher percentage of unsaturated C18 fatty acids than other lipid compositions. However, as they transition from free-living to the parasitic stage, considerable changes occur, including alterations in energy metabolism (Harder, 2016) and membrane fatty acid composition (Proudfoot et al., 1990). For instance, free-living stages require more unsaturated fatty acids in their membrane to protect themselves from low environmental temperatures, but these fatty acids are not required as they develop into the parasitic stage (Hazel & Williams, 1990). The majority of the fatty acids identified in adult stages of *A. caninum* (Wangchuk et al., 2019c), *T. muris* and *N. brasiliensis* (Wangchuk et al., 2019b), *D. caninum* (Wangchuk et al., 2019a), and *D. viviparus* (Becker et al., 2017) were saturated fatty acids, which supports the phospholipid membrane hypothesis (Proudfoot et al., 1990), as membrane phospholipids have a polar head and two nonpolar tails (composed of fatty acids), whereby one of the tails has saturated fatty acids (Lombard, 2014). However, in *Haemonchus contortus*, fatty acid saturation levels decreased as they matured into the parasitic stage (Wang et al., 2018). There was a reduced synthesis of triradylglycerols and increased glycerophospholipids (predominantly glycerophosphoethanolamines and glycerophosphocholines) as *H. contortus* transitioned from free-living to the parasitic stage. In *Trichinella papuae* L1 stage, glycerophospholipids were dominant, with the most abundant glycerolipid diglycerides (Mangmee et al., 2020). The tegumental membranes of *S. mansoni* are also enriched with unsaturated fatty acids such as eicosenoic acid (20:1) and 5-octadecenoic acid, which were absent in their host (Retra et al., 2015). Phosphatidylethanolamines (PE) were abundant in *O. ochengi* worms and bovine nodule fluid, suggesting that these phospholipids might be released from *O. ochengi* into the host and could serve as potential biomarkers (Wewer et al., 2017).

Giera et al. (2018) found that the lipid composition of different life cycle stages of *S. mansoni* varied. Prostaglandins were enriched in eggs, while the cercaria stage contains mainly resolvins. Mature eggs of *S. mansoni* had higher levels of phospholipids, while immature eggs contained more neutral lipids (Bexkens et al., 2019). *S. mansoni* does not oxidise fatty acids because they lack the genes encoding enzymes required for β-oxidation. Instead, they (mostly females) uptake and use fatty acids, including stored fatty acids such as triacylglycerols, which are used for membrane phospholipid biosynthesis in developing miracidia (Bexkens et al., 2019). Prostaglandins are not only enriched in *S. mansoni* eggs, but they are also major lipids in *T. suis* ESPs (Laan et al., 2017).

## Metabolic pathways and biosynthesis

This review found that 155 metabolites, including 100 polar compounds and 55 lipids were identified using MSI level-1 identification protocols. Therefore, our discussion of biosynthetic pathways is focussed on these metabolites. We observed that helminths primarily rely on amino acids, carbohydrates, and lipids metabolism in both adults and infective stages, depending on the culture media used for ESPs collection. Amino acids were the most commonly reported metabolites from helminths, and pathway analysis revealed that aminoacyl-tRNA biosynthesis, arginine biosynthesis, lysine degradation, aspartate, alanine, and glutamate metabolism were the most common amino acid pathways (Yeshi et al., 2020). Yeshi et al. (2020) also suggested that isocitrate, a unique metabolite in the ESPs of *N. brasiliensis* infective stage, could be the product of glyoxylate metabolism, a common pathway in free-living nematodes. Adult helminth parasites typically use two forms of energy metabolism: anaerobic glycolysis, which is predominant in schistosomes and filarial nematodes, and degradation of carbohydrates to phosphoenolpyruvate (PEP) through the same glycolytic pathway without oxygen (Tielens, 1994). The presence of PEP in adult *T. muris* and most of the helminths included in this review suggests that anaerobic degradation of carbohydrates to PEP could be one of the primary energy pathways.

Parasites typicaally have a functionally incomplete TCA cycle (Prichard, 1989), as observed in the parasitic helminths discussed in this review. Glucose is obtained from the host and subsequently utilised in anaerobic glycolysis (Müller et al., 2012). Parasitic helminths, in particular, prefer lipids and amino acids over carbohydrates as a source of energy (Clark, 1969). Amino acid metabolism is a major pathway for these parasites, and they use the TCA cycle and fatty acid degradation for this purpose rather than for ATP production (Tielens and van den Bergh, 1993). Studies have shown that C^14^-labelled glucose in dog hookworm is not converted to glycogen (Araujo et al., 2013) but is instead diverted into amino acid production (Perez Gimenez et al., 1967) in a metabolism dominated by fermentative processes (Müller et al., 2012; Warren & Poole, 1970). Exposure to dog sera increases feeding rates in dog hookworm (L3 stage) (Warren & Guevara, 1962), and large diffusible solutes (such as the protein fraction) stimulate glucose consumption (Komiya et al., 1956). Adult dog hookworms are capable of aerobic metabolism, and cyanide inhibition studies indicate that they have a TCA cycle that can oxidize pyruvic and succinic acids (Warren & Karlsson, 1965). However, NADH respiration is not strongly coupled to oxidative phosphorylation, and hookworms lack respiratory control (Warren, 1970). The activity of succinoxidase in adult *A. caninum* is not tightly coupled to the synthesis of ATP, and external NADH oxidation that is not coupled to phosphorylation can occur. The low phosphorylation or oxidation ratios may reflect loosely coupled respiratory pathways or the existence of two respiration pathways—one coupled to the esterification of inorganic phosphate and another to the NADH pathway (Warren, 1970).

Recent studies have shown significant interest in immunometabolism, which investigates the metabolic profiles of activated immune cells and their role in immune homeostasis (O'Neill et al., 2016). In particular, six pathways have been linked to immune function: glycolysis (which is pro-inflammatory), the TCA cycle, the pentose phosphate pathway, fatty acid oxidation, fatty acid synthesis, and amino acid metabolism. Interestingly, high levels of fatty acids and amino acids have been found to inhibit cell activation of the mTOR pathway, which can have anti-inflammatory effects (O'Neill et al., 2016). It would be intriguing to investigate the levels of mTOR expression at the site of gastrointestinal helminth attachment in the gut to determine whether this is one of the mechanisms by which the worms induce immune tolerance. Furthermore, M1 and M2 macrophages have distinct differences in their TCA cycles, with M2 macrophages having a complete TCA cycle, while M1 macrophages have a TCA cycle that is broken in two places (after citrate and succinate) (O'Neill et al., 2016). Notably, M2 macrophages are associated with helminth expulsion, yet we observe that *A. caninum* exhibits the broken TCA cycle pattern associated with M1 macrophages, suggesting that other factors may influence macrophage polarisation in helminth infections.

The interactions of the end products of helminth metabolism within the host have not been extensively studied. Additionally, there needs to be more investigation into the role of secondary metabolites produced by helminths and their roles in the host-parasite relationship. Parasites often utilize fermentation as a metabolism, producing end products such as short-chain fatty acids (SCFAs). However, we can easily distinguish cellular intermediates of biochemical pathways in the case of *A. caninum*. While these molecules may be released from dying cells or the worm itself, we believe that the hookworm actively secretes these molecules to create an environment of tolerance and immune homeostasis around its attachment sites, despite the relative impermeability of its outer cuticle. It has been observed that successful parasites, including *A. caninum*, use anaerobic metabolism during active parasitism to produce fatty acids, such as SCFAs, which may play essential roles in modulating host immune responses.

Helminths in their parasitic stages contain more diverse range of fatty acids than their non-parasitic stages (Becker et al., 2017), as lipids are seential for establishing their niches inside their hosts (Sato et al., 2008). Glucose is metabolized to produce SCFAs, such as acetate and propionate, while L-valine and L-leucine are the precursors of isobutyric acid and isovaleric acid, respectively, as demonstrated by labelling studies (Warren, 1970). *A. lumbricoides* was the first to have a few SCFAs, such as acetic acid, propionic acid, *n*-valeric acid, methylbutyric acid, and methylvaleric acid, reported in 1965 (Beames, 1965). Our studies involving *A. caninum*, *D. caninum*, *T. canis*, and *T. muris* (Wangchuk et al., 2019b, 2019c) showed that fatty acids, including SCFAs, were the major lipids metabolised when cultured outside their host using a single-component culture media (Glutamax). Another group reported the presence of saturated fatty acids (not SCFAs) from the ova of *A. caninum* (Gyawali et al., 2016). The origin of the excreted SCFAs of *A. caninum* was demonstrated through D-glucose-^14^C isotope labelling, which suggested intermediary glucose metabolism in both aerobes and anaerobes (Warren & Poole, 1970). The formation and excretion of acetate as a metabolic end-product of energy metabolism have also been reported in many other helminth parasites, such as *F. hepatica*, *A. suum,* and *H. contortus* (Tielens et al., 2010).

However, another body of scholarly literature suggests that helminths cannot synthesize most essential lipids, including SCFAs, and instead rely on obtaining them exogenously from their host (Smyth, 1994). For instance, it is reported that schistosomes obtain lipids from their host and convert them to triglyceride (TG) (Brouwers et al., 1997), as they cannot synthesize fatty acids (Berriman et al., 2009). Analyses of the available literature have revealed that two enzymes are involved in propanoate synthesis in *A. caninum*. However, enzymes involved in SCFAs biosynthesis, such as cytosolic acetyl-CoA synthetase or an organellar acetate: succinate CoA-transferase, are poorly represented when mapped against the known metabolic KEGG pathways of 81 worm genomes (Wangchuk et al., 2019c). This suggests that helminths are unable to synthesize SCFAs. It has been suggested that gut microbiota could be another source of SCFAs in helminth ESPs. Studies have shown that experimental human hookworm infection enriches bacterial species in the gut interface and elevates the production of SCFAs (Giacomin et al., 2015). The SCFAs such as acetate, butyrate, and propionate are produced and utilized by bacteria and benefit host epithelial cells by producing molecules such as vitamin B_12_ (Belzer et al., 2017). However, due to contradictory biosynthetic information described in the literature, further studies will be needed to define the contribution of the commensal microbiome to fatty acid production, especially SCFAs synthesis in helminths.

Helminth-derived lipids also participate in biochemical interactions between the host and the parasite. However, most lipids reported from various helminth metabolomics studies, including those listed in Table 2, are putative, and only about 55 lipids have been identified at MSI level-1 (confirmed with reference standards), primarily fatty acyls (as shown in Table 3). Fatty acids are crucial in various biological processes, and some, such as the *cis*-form of octadecanoic acid (stearic acid), monoenoic acids (oleic acid and vaccenic acid), and other branched-chain acids, aid in penetrating the host cell membrane (Ward, 1982). The presence of octadecanoic acid (stearic acid) and oleic acid in the ESPs or somatic tissues of at least seven helminths (as listed in Table 1) suggests that these compounds may play a role in host invasion and establishing infection (Yeshi et al., 2020).

The utilization of lipids by helminths varies across different stages of their life-cycle. During the parasitic adult stage, helminths mainly rely on their host for energy and nutrients, utilizing only specific fatty acids and fat-soluble vitamins for energy (Tielens, 1997). Many lipids are stored and excreted, later becoming food for gametes in adults, without other essential functions (Barrett, 1968; Cheng, 1986). It is hypothesized that SCFAs, such as propionate and acetate, identified in the ESPs of adult *A. caninum*, *N. brasiliensis*, and *T. muris*, may be stored as food reserves for gametes (Andoh et al., 1999; Kovarik et al., 2011; Tedelind et al., 2007). Other lipids, such as phosphatidylcholines (PCs), have been identified as major lipids in *S. mansoni* (Ferreira et al., 2015), while pterin was detected in the ESPs of the infective L3 stage of *N. brasiliensis* (Yeshi et al., 2020). Succinate (succinic acid) is among the metabolites reported to be secreted or excreted by a few helminth species, including *N. brasiliensis, N. americanus, T. muris, A. caninum, D. caninum,* and *T. canis* (Table 1).

Over 500 putative metabolites, including lipids, have been reported from a single helminth species (Wang et al., 2018, 2020; Wangchuk et al., 2019b; Yeshi et al., 2020), yet their bioactivity remains largely unexplored. Currently, only 20–30% of the total metabolites are known, with 70–80% remaining unidentified due to limitations in identification protocols and helminth-specific compound libraries. There is a pressing need for extensive and comprehensive metabolomics studies. In 2020, a new pulsed MS ion generation technique called triboelectric nanogenerator inductive nanoelectrospray ionization (TENGi nanoESI) MS was introduced. This technique enables the analysis of volume-limited samples, even at nanolitre scale, via LC–MS (Li et al., 2020). Such technological advancement are likely to popularise metabolomics studies of helminths. Isolating compounds from helminths could result in discovering many novel molecules new to science, thereby improving the understanding of helminth biology and their molecular interactions with hosts.

## Conclusion

While there is a significant amount of literature on genomic and proteomic analyses of parasitic helminths, metabolomics is a relatively new ‘omics’ technology that has recently been applied to study helminth metabolomics. The metabolome is the final downstream product of the genome and proteome and has proven to be complementary approach to genomics and proteomics techniques in understanding helminth biology at a more comprehensive level. Metabolomics techniques are increasingly being used to study helminths in various ways, including in vitro parasite culture, in vivo animal models, and clinical studies in humans. Initially, many metabolomics studies focused on detecting changes in the metabolome profile of infected hosts (mostly in vivo animal and human biofluids) compared to ESPs derived from in vitro parasite culture.

We analyzed 28 studies that reported the metabolomic assessment of ESPs and somatic tissue extracts of 17 helminth species grown under in vitro culture conditions. Of these 28 reported studies, included in this review, 19 achieved the highest level of metabolite identification (MSI level-1), while the remaining studies reported MSI level-2 identification. Only 155 small molecule metabolites, including polar and lipids, were identified using MSI level-1 characterization protocols from various helminth species. Although MSI level-1 is the best and the highest identification level, its use is limited by the number of known standards, which are often expensive and may not be readily available to researchers or metabolomics institutes. As a result, targeted and MSI level-1 platforms are only sometimes feasible options for researchers.

The advances in analytical technologies and identification tools offer immense potential for providing a comprehensive metabolic snapshot of helminths throughout their lifecycle and greater opportunities for higher identification rates. However, several challenges must be addressed when using this latest ‘omics’ platform. These challenges include:

- The need for expensive analytical instrumentation such as MS and NMR to obtain raw data makes it challenging for resourced-constrained countries with high helminth endemicity to conduct metabolomics studies. Moreover, none of these analytical platforms can provide a complete picture of complex helminth-derived samples.
- The requirement of sophisticated bioinformatics tools and statistical software for data management, integration, mining, and interpretation. Although many free software programs are available, no standardized software programs can be used across all the analytical platforms.
- Existing helminth databases, such as WormBook, WormBase, and Wormatlas focus on the biology, breeding, genome, proteome, and biochemistry of the free-living model nematode *Caenorhabditis elegans*, rather than parasitic helminths. As the metabolic pathways and metabolome compositions of the free-living *C. elegans* and parasitic helminths are expected to be different, a database specializing in parasitic helminth-specific small molecules is needed.

## References

[CR1] Andoh A, Bamba T, Sasaki M (1999). Physiological and anti-inflammatory roles of dietary fiber and butyrate in intestinal functions. Journal of Parenteral and Enteral Nutrition.

[CR2] Araujo A, Reinhard K, Ferreira LF, Pucu E, Chieffi PP (2013). Paleoparasitology: The origin of human parasites. Arquivos De Neuro-Psiquiatria.

[CR3] Barrett J (1968). Lipids of the infective and parasitic stages of some nematodes. Nature.

[CR4] Barrett J (1981). Biochemistry of parasitic helminths.

[CR5] Barrett J (1987). Developmental aspects of metabolism in parasites. International Journal for Parasitology.

[CR6] Beames CG (1965). Neutral lipids of *Ascaris lumbricoides* with special reference to the esterified fatty acids. Experimental Parasitology.

[CR7] Becker A-C, Willenberg I, Springer A, Schebb NH, Steinberg P, Strube C (2017). Fatty acid composition of free-living and parasitic stages of the bovine lungworm *Dictyocaulus viviparus*. Molecular and Biochemical Parasitology.

[CR8] Belzer C, Chia LW, Aalvink S, Chamlagain B, Piironen V, Knol J, de Vos WM (2017). Microbial metabolic networks at the mucus layer lead to diet-independent butyrate and vitamin B12 production by intestinal symbionts. Mbio.

[CR9] Berriman M, Haas BJ, LoVerde PT, Wilson RA, Dillon GP, Cerqueira GC, Mashiyama ST, Al-Lazikani B, Andrade LF, Ashton PD, Aslett MA, Bartholomeu DC, Blandin G, Caffrey CR, Coghlan A, Coulson R, Day TA, Delcher A, DeMarco R, Djikeng A, Eyre T, Gamble JA, Ghedin E, Gu Y, Hertz-Fowler C, Hirai H, Hirai Y, Houston R, Ivens A, Johnston DA, Lacerda D, Macedo CD, McVeigh P, Ning Z, Oliveira G, Overington JP, Parkhill J, Pertea M, Pierce RJ, Protasio AV, Quail MA, Rajandream MA, Rogers J, Sajid M, Salzberg SL, Stanke M, Tivey AR, White O, Williams DL, Wortman J, Wu W, Zamanian M, Zerlotini A, Fraser-Liggett CM, Barrell BG, El-Sayed NM (2009). The genome of the blood fluke *Schistosoma mansoni*. Nature.

[CR10] Bexkens ML, Mebius MM, Houweling M, Brouwers JF, Tielens AGM, van Hellemond JJ (2019). Schistosoma mansoni does not and cannot oxidise fatty acids, but these are used for biosynthetic purposes instead. International Journal for Parasitology.

[CR11] Brouwers JF, Smeenk IM, van Golde LM, Tielens AG (1997). The incorporation, modification and turnover of fatty acids in adult *Schistosoma mansoni*. Molecular and Biochemical Parasitology.

[CR12] Chen Y, Zhang M, Ding X, Yang Y, Chen Y, Zhang Q, Fan Y, Dai Y, Wang J (2021). Mining anti-Inflammation molecules from *Nippostrongylus brasiliensis*-derived products through the metabolomics approach. Frontiers in Cellular and Infection Microbiology.

[CR13] Cheng TC (1986). General Parasitology.

[CR14] Clark FE (1969). *Ancylostoma caninum*: Food reserves and changes in chemical composition with age in third stage larvae. Experimental Parasitology.

[CR300] Dettmer K, Aronov PA, Hammock BD (2007). Mass spectrometry-based metabolomics. Mass Spectrometry Reviews.

[CR15] Eichenberger RM, Talukder MH, Field MA, Wangchuk P, Giacomin P, Loukas A, Sotillo J (2018). Characterization of *Trichuris muris* secreted proteins and extracellular vesicles provides new insights into host–parasite communication. Journal of Extracellular Vesicles.

[CR16] Elbein AD, Pan YT, Pastuszak I, Carroll D (2003). New insights on trehalose: A multifunctional molecule. Glycobiology.

[CR17] Fairbairn D (1958). Glucose, trehalose and glycogen in *Porrocaecum decipiens* Larvæ. Nature.

[CR18] Fairbairn D (1958). Trehalose and glucose in helminths and other invertebrates. Canadian Journal of Zoology.

[CR19] Fairbairn D, Passey RF (1957). Occurrence and distribution of trehalose and glycogen in the eggs and tissues of *Ascaris lumbricoides*. Experimental Parasitology.

[CR20] Ferreira MS, de Oliveira DN, de Oliveira RN, Allegretti SM, Catharino RR (2014). Screening the life cycle of *Schistosoma mansoni* using high-resolution mass spectrometry. Analytica Chimica Acta.

[CR21] Ferreira MS, de Oliveira DN, de Oliveira RN, Allegretti SM, Vercesi AE, Catharino RR (2014). Mass spectrometry imaging: A new vision in differentiating *Schistosoma mansoni* strains. Journal of Mass Spectrometry.

[CR22] Ferreira MS, de Oliveira RN, de Oliveira DN, Esteves CZ, Allegretti SM, Catharino RR (2015). Revealing praziquantel molecular targets using mass spectrometry imaging: An expeditious approach applied to *Schistosoma mansoni*. International Journal for Parasitology.

[CR23] Fukushima T (1970). Biopterin: Reduced form in *Ascaris lumbricoides*. Experimental Parasitology.

[CR24] Ghosh A, Kar K, Ghosh D, Dey C, Misra KK (2010). Major lipid classes and their fatty acids in a parasitic nematode, *Ascaridia galli*. Journal of Parasitic Diseases.

[CR25] Giacomin P, Zakrzewski M, Croese J, Su X, Sotillo J, McCann L, Navarro S, Mitreva M, Krause L, Loukas A, Cantacessi C (2015). Experimental hookworm infection and escalating gluten challenges are associated with increased microbial richness in celiac subjects. Science and Reports.

[CR26] Giera M, Kaisar MMM, Derks RJE, Steenvoorden E, Kruize YCM, Hokke CH, Yazdanbakhsh M, Everts B (2018). The *Schistosoma mansoni* lipidome: Leads for immunomodulation. Analytica Chimica Acta.

[CR27] Ginger CD, Fairbairn D (1966). Lipid metabolism in helminth parasites. I. The lipids of *Hymenolepis diminuta* (cestoda). Journal of Parasitology.

[CR28] Greichus A, Greichus YA (1966). Chemical composition and volatile fatty acid production of male *Ascaris lumbricoides* before and after starvation. Experimental Parasitology.

[CR29] Gyawali P, Beale DJ, Ahmed W, Karpe AV, Magalhaes RJ, Morrison PD, Palombo EA (2016). Determination of *Ancylostoma caninum* ova viability using metabolic profiling. Parasitology Research.

[CR30] Harder A (2016). The biochemistry of *Haemonchus contortus* and other parasitic nematodes. Advances in Parasitology.

[CR31] Hazel JR, Williams EE (1990). The role of alterations in membrane lipid composition in enabling physiological adaptation of organisms to their physical environment. Progress in Lipid Research.

[CR32] Hopkins FG (1894). The pigments of the pieridae. A contribution to the study of excretory substances which function in ornament. Proceedings of the Royal Society of London.

[CR33] Hotez PJ, Brindley PJ, Bethony JM, King CH, Pearce EJ, Jacobson J (2008). Helminth infections: The great neglected tropical diseases. The Journal of Clinical Investigation.

[CR34] Joachim A, Ryll M, Daugschies A (2000). Fatty acid patterns of different stages of *Oesophagostomum dentatum* and *Oesophagostomum quadrispinulatum* as revealed by gas chromatography. International Journal for Parasitology.

[CR35] Kadesch P, Quack T, Gerbig S, Grevelding CG, Spengler B (2020). Tissue- and sex-specific lipidomic analysis of *Schistosoma mansoni* using high-resolution atmospheric pressure scanning microprobe matrix-assisted laser desorption/ionization mass spectrometry imaging. PLoS Neglected Tropical Diseases.

[CR36] Kalf GF, Rieder SV (1958). The purification and properties of trehalase. Journal of Biological Chemistry.

[CR37] Kokova D, Mayboroda OA (2019). Twenty years on: Metabolomics in helminth research. Trends in Parasitology.

[CR38] Komiya Y, Yasuraoka K, Sato A (1956). Survival of *Ancylostoma caninum* in vitro I. Japanese Journal of Medical Science and Biology.

[CR39] Kovarik JJ, Tillinger W, Hofer J, Hölzl MA, Heinzl H, Saemann MD, Zlabinger GJ (2011). Impaired anti-inflammatory efficacy of n-butyrate in patients with IBD. European Journal of Clinical Investigation.

[CR40] Laan LC, Williams AR, Stavenhagen K, Giera M, Kooij G, Vlasakov I, Kalay H, Kringel H, Nejsum P, Thamsborg SM, Wuhrer M, Dijkstra CD, Cummings RD, Van Die I (2017). The whipworm (*Trichuris suis*) secretes prostaglandin E2 to suppress proinflammatory properties in human dendritic cells. FASEB Journal.

[CR41] Learmonth MP, Euerby MR, Jacobs DE, Gibbons WA (1987). Metabolite mapping of *Toxocara canis* using one- and two-dimensional proton magnetic resonance spectroscopy. Molecular and Biochemical Parasitology.

[CR42] Li Y, Bouza M, Wu C, Guo H, Huang D, Doron G, Temenoff JS, Stecenko AA, Wang ZL, Fernández FM (2020). Sub-nanoliter metabolomics via mass spectrometry to characterize volume-limited samples. Nature Communications.

[CR43] Loffler M, Carrey EA, Zameitat E (2016). Orotate (orotic acid): An essential and versatile molecule. Nucleos Nucleot Nucl.

[CR44] Lombard J (2014). Once upon a time the cell membranes: 175 years of cell boundary research. Biology Direct.

[CR45] Loukas A, Hotez PJ, Diemert D, Yazdanbakhsh M, McCarthy JS, Correa-Oliveira R, Croese J, Bethony JM (2016). Hookworm Infection. Nature Reviews Disease Primers.

[CR46] Maizels RM, Smits HH, McSorley HJ (2018). Modulation of host immunity by helminths: The expanding repertoire of parasite effector molecules. Immunity.

[CR47] Mangmee S, Adisakwattana P, Tipthara P, Simanon N, Sonthayanon P, Reamtong O (2020). Lipid profile of *Trichinella papuae* muscle-stage larvae. Science and Reports.

[CR48] McSorley HJ, Hewitson JP, Maizels RM (2013). Immunomodulation by helminth parasites: Defining mechanisms and mediators. International Journal of Parasitology.

[CR49] Melo CF, Esteves CZ, de Oliveira RN, Guerreiro TM, de Oliveira DN, Lima EO, Mine JC, Allegretti SM, Catharino RR (2016). Early developmental stages of *Ascaris lumbricoides* featured by high-resolution mass spectrometry. Parasitology Research.

[CR50] Minematsu T, Yamazaki S, Uji Y, Okabe H, Korenaga M, Tada I (1990). Analysis of polyunsaturated fatty acid composition of *Strongyloides ratti* in relation to development. Journal of Helminthology.

[CR51] Moran M, Moran M (2011). Global funding of new products for neglected tropical diseases. The Causes and Impacts of Neglected Tropical and Zoonotic Diseases: Opportunities for Integrated Intervention Strategies.

[CR52] Müller M, Mentel M, van Hellemond JJ, Henze K, Woehle C, Gould SB, Yu RY, van der Giezen M, Tielens AG, Martin WF (2012). Biochemistry and evolution of anaerobic energy metabolism in eukaryotes. Microbiology and Molecular Biology Reviews.

[CR53] Nadjsombati MS, McGinty JW, Lyons-Cohen MR, Jaffe JB, DiPeso L, Schneider C, Miller CN, Pollack JL, Nagana Gowda GA, Fontana MF, Erle DJ, Anderson MS, Locksley RM, Raftery D, von Moltke J (2018). Detection of succinate by intestinal tuft cells triggers a type 2 innate immune circuit. Immunity.

[CR54] Nishina M, Matsushita K, Furuhata T, Hori E, Takahashi M, Kato K, Matsuda H (1994). Nuclear magnetic resonance (NMR) analysis on the serum-lipids of rabbits infected with *Schistosoma japonicum*—Oxidative modifications of Diene system in fatty chains. International Journal for Parasitology.

[CR55] O'Neill LA, Kishton RJ, Rathmell J (2016). A guide to immunometabolism for immunologists. Nature Reviews Immunology.

[CR56] Perez de Souza L, Alseekh S, Scossa F, Fernie AR (2021). Ultra-high-performance liquid chromatography high-resolution mass spectrometry variants for metabolomics research. Nature Methods.

[CR57] Perez Gimenez ME, Gimenez A, Gaede K (1967). Metabolic transformation of 14C-glucose into tissue proteins of *Ancylostoma caninum*. Experimental Parasitology.

[CR58] Preidis GA, Hotez PJ (2015). The newest “omics”–metagenomics and metabolomics–enter the battle against the neglected tropical diseases. PLoS Neglected Tropical Diseases.

[CR59] Prichard RK, Bennet E-M, Behm C, Bryant C (1989). How do Parasitic Helminths use and Survive Oxygen and Oxygen Metabolites. Comparative Biochemistry of Parasitic Helminths.

[CR60] Proudfoot L, Kusel JR, Smith HV, Harnett W, Worms MJ, Kennedy MW (1990). The surface lipid of parasitic nematodes: Organization, and modifications during transition to the mammalian host environment. Acta Tropica.

[CR61] Retra K, deWalick S, Schmitz M, Yazdanbakhsh M, Tielens AG, Brouwers JF, van Hellemond JJ (2015). The tegumental surface membranes of Schistosoma mansoni are enriched in parasite-specific phospholipid species. International Journal for Parasitology.

[CR62] Ritler D, Rufener R, Li JV, Kämpfer U, Müller J, Bühr C, Schürch S, Lundström-Stadelmann B (2019). In vitro metabolomic footprint of the *Echinococcu multilocularis* metacestode. Science and Reports.

[CR63] Ryan SM, Eichenberger RM, Ruscher R, Giacomin PR, Loukas A (2020). Harnessing helminth-driven immunoregulation in the search for novel therapeutic modalities. PLoS Pathogens.

[CR64] Sanchez AL, Gabrie JA, Rueda MM, Mejia RE, Bottazzi ME, Canales M (2014). A scoping review and prevalence analysis of soil-transmitted helminth infections in Honduras. PLoS Neglected Tropical Diseases.

[CR65] Sato S, Hirayama T, Hirazawa N (2008). Lipid content and fatty acid composition of the monogenean *Neobenedenia girellae* and comparison between the parasite and host fish species. Parasitology.

[CR66] Shen R, Zhang Y, Ayling JE, Nair MG, Baugh CM (1993). Reduced pterins as scavengers for reactive oxygen species. Chemistry and Biology of Pteridines and Folates.

[CR67] Smith VP, Selkirk ME, Gounaris K (1996). Identification and composition of lipid classes in surface and somatic preparationss of adult *Brugia malayi*. Molecular and Biochemical Parasitology.

[CR68] Smyth JD (1994). Animal Parasitology.

[CR69] Tedelind S, Westberg F, Kjerrulf M, Vidal A (2007). Anti-inflammatory properties of the short-chain fatty acids acetate and propionate: A study with relevance to inflammatory bowel disease. World Journal of Gastroenterology.

[CR70] Tielens AGM (1994). Energy generation in parasitic helminths. Parasitology Today.

[CR71] Tielens AGM, Fried B, Graczyk TK (1997). Biochemistry of trematodes. Advances in trematode biology.

[CR72] Tielens AGM, van den Bergh SG, Hodachka PW, Lutz PL, Sick T, Rosenthal M, van den Thillart G (1993). Aerobic and Anaerobic energy metabolism in the lifecycle of parasitic helminths. Surviving hypoxia: Mechanisms of control and adaption.

[CR73] Tielens AG, van Grinsven KW, Henze K, van Hellemond JJ, Martin W (2010). Acetate formation in the energy metabolism of parasitic helminths and protists. International Journal for Parasitology.

[CR74] Wang T, Nie S, Ma G, Korhonen PK, Koehler AV, Ang CS, Reid GE, Williamson NA, Gasser RB (2018). The developmental lipidome of *Haemonchus contortus*. International Journal for Parasitology.

[CR75] Wang T, Nie S, Ma G, Vlaminck J, Geldhof P, Williamson NA, Reid GE, Gasser RB (2020). Quantitative lipidomic analysis of *Ascaris suum*. PLoS Neglected Tropical Diseases.

[CR76] Wangchuk P, Anderson D, Yeshi K, Loukas A (2021). Identification of small molecules of the infective stage of human hookworm using LCMS-based metabolomics and lipidomics protocols. ACS Infectious Diseases.

[CR77] Wangchuk P, Constantinoiu C, Eichenberger RM, Field M, Loukas A (2019). Characterization of tapeworm metabolites and their reported biological activities. Molecules.

[CR78] Wangchuk P, Kouremenos K, Eichenberger RM, Pearson M, Susianto A, Wishart DS, McConville MJ, Loukas A (2019). Metabolomic profiling of the excretory-secretory products of hookworm and whipworm. Metabolomics.

[CR79] Wangchuk P, Lavers O, Wishart DS, Loukas A (2020). Excretory/secretory metabolome of the zoonotic roundworm parasite *Toxocara canis*. Biomolecules.

[CR80] Wangchuk P, Shepherd C, Constantinoiu C, Ryan RYM, Kouremenos KA, Becker L, Jones L, Buitrago G, Giacomin P, Wilson D, Daly N, McConville MJ, Miles JJ, Loukas A (2019). Hookworm-derived metabolites suppress pathology in a mouse model of colitis and inhibit secretion of key inflammatory cytokines in primary human leukocytes. Infection and Immunity.

[CR81] Ward PF (1982). Aspects of helminth metabolism. Parasitology.

[CR82] Warren LG (1970). Biochemistry of the dog hookworm III. Oxidative phosphorylation. Experimental Parasitology.

[CR83] Warren LG, Guevara A (1962). Nematode metabolism with special reference to *Ancylostoma caninum*. Revista De Biologia Tropical.

[CR84] Warren LG, Karlsson EL (1965). Biochemistry of the dog hookworm I. Oxidative Metabolism. Experimental Parasitology.

[CR85] Warren LG, Poole WJ (1970). Biochemistry of the dog hookworm. II. Nature and origin of the excreted fatty acids. Experimental Parasitology.

[CR86] Weiss G, Fuchs D, Hausen A, Reibnegger G, Werner ER, Werner-Felmayer G, Semenitz E, Dierich MP, Wachter H (1993). Neopterin modulates toxicity mediated by reactive oxygen and chloride species. FEBS Journal.

[CR87] Wewer V, Makepeace BL, Tanya VN, Peisker H, Pfarr K, Hoerauf A, Dormann P (2017). Lipid profiling of the filarial nematodes *Onchocerca volvulus*, *Onchocerca ochengi* and *Litomosoides sigmodontis* reveals the accumulation of nematode-specific ether phospholipids in the host. International Journal for Parasitology.

[CR88] Whitman JD, Sakanari JA, Mitreva M (2021). Areas of metabolomic exploration for helminth infections. ACS Infectious Diseases.

[CR89] WHO (2020) Soil-transmitted helminth infections, World Health Organization.

[CR90] Yeshi K, Creek DJ, Anderson D, Ritmejerytė E, Becker L, Loukas A, Wangchuk P (2020). Metabolomes and lipidomes of the infective stages of the gastrointestinal nematodes, *Nippostrongylus brasiliensis* and *Trichuris muris*. Metabolites.

[CR91] Yeshi K, Ruscher R, Loukas A, Wangchuk P (2022). Immunomodulatory and biological properties of helminth-derived small molecules: Potential applications in diagnostics and therapeutics. Frontiers in Parasitology.

[CR92] Zakeri A, Hansen EP, Andersen SD, Williams AR, Nejsum P (2018). Immunomodulation by helminths: Intracellular pathways and extracellular vesicles. Frontiers in Immunology.

